# Lanthanoid Luminophores with Linear, Bipyridine-Based Antenna Ligands

**DOI:** 10.1021/acs.inorgchem.5c03839

**Published:** 2025-10-28

**Authors:** Christian Kruck, Timo Neumann, Alexander Schäfer, Elisabeth Kreidt, Patrick Weis, Christof Holzer, Manfred M. Kappes, Michael Seitz

**Affiliations:** † Institute of Inorganic Chemistry, University of Tübingen, Auf der Morgenstelle 18, 72076 Tübingen, Germany; ‡ Institute of Physical Chemistry, Karlsruhe Institute of Technology (KIT), 76131 Karlsruhe, Germany; § Institute of Nanotechnology, Karlsruhe Institute of Technology (KIT), 76344 Eggenstein-Leopoldshafen, Germany; ∥ Department of Chemistry and Chemical Biology, TU Dortmund, Otto-Hahn-Str. 6, 44227 Dortmund, Germany; ⊥ Institute for Quantum Materials and Technologies, Karlsruhe Institute of Technology (KIT), 76131 Karlsruhe, Germany

## Abstract

A new, linear octadentate chelator, “en-pypa,” based on 2,2′-bipyridine-6-carboxylic acid, has been developed. This ligand can bind trivalent lanthanoids (e.g., Sm, Eu, Tb, Dy, Tm, Yb, and Lu) very rapidly and yields well-defined complexes that exhibit relatively strong luminescence in aqueous solution. This study reports the synthesis, as well as the structural and photophysical characterization. In addition, nonradiative deactivation of near-infrared luminescence by the ligand N–H oscillators is addressed by comparison of the luminescence from the Yb complexes of en-pypa and its methylated analogue.

## Graphical abstract

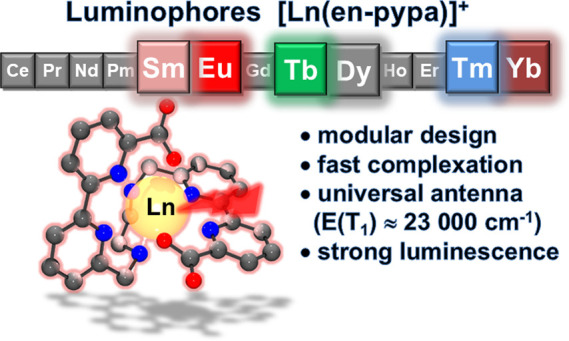

## Introduction

Lanthanoid luminophores continue to attract considerable attention due to their wide applicability and their often very advantageous photophysical properties.^1^ One problem, however, that has to be solved for luminescence applications is the difficulty to populate lanthanoid excited states. Direct, f-f-absorption is parity forbidden and the long-standing strategy to circumvent this problem is to sensitize luminescence by an “antenna” ligand. The latter, if designed appropriately, can transfer energy onto emitting lanthanoid states from a ligand triplet state after photoexcitation. One of the crucial parameters in this sensitization scheme is the energetic gap between the feeding triplet and the lanthanoid accepting state. Since the latter is different for every lanthanoid it is rare that a specific antenna moiety can efficiently be used for more than one or two different lanthanoids. Among the most successful ligand building blocks in coordination chemistry overall and, more particularly, for the use as antennae in lanthanoid luminescence are 2,2′-bipyridine-based units.^2^ One of the most iconic examples featuring these building blocks are Lehn’s macrobicyclic, tris­(2,2′-bipyridine) cryptates **1-Ln** (Figure 1) and related architectures.^3^ Another very successful bipyridine sensitizer is the relatively simple 2,2′-bipyridine-6,6′-dicarboxylate which forms homoleptic 2:1 complexes **2-Ln** (Figure 1). These ligand architectures are universal lanthanoid antennae and have allowed to sensitize luminescence from almost all relevant lanthanoids.^4^ Close multidentate relatives of **2-Ln** are the lanthanoid complexes exemplified by **3-Ln** (Figure 1) featuring 2,2′-bipyridine-6-carboxylate motifs.^5^

**Figure 1:**
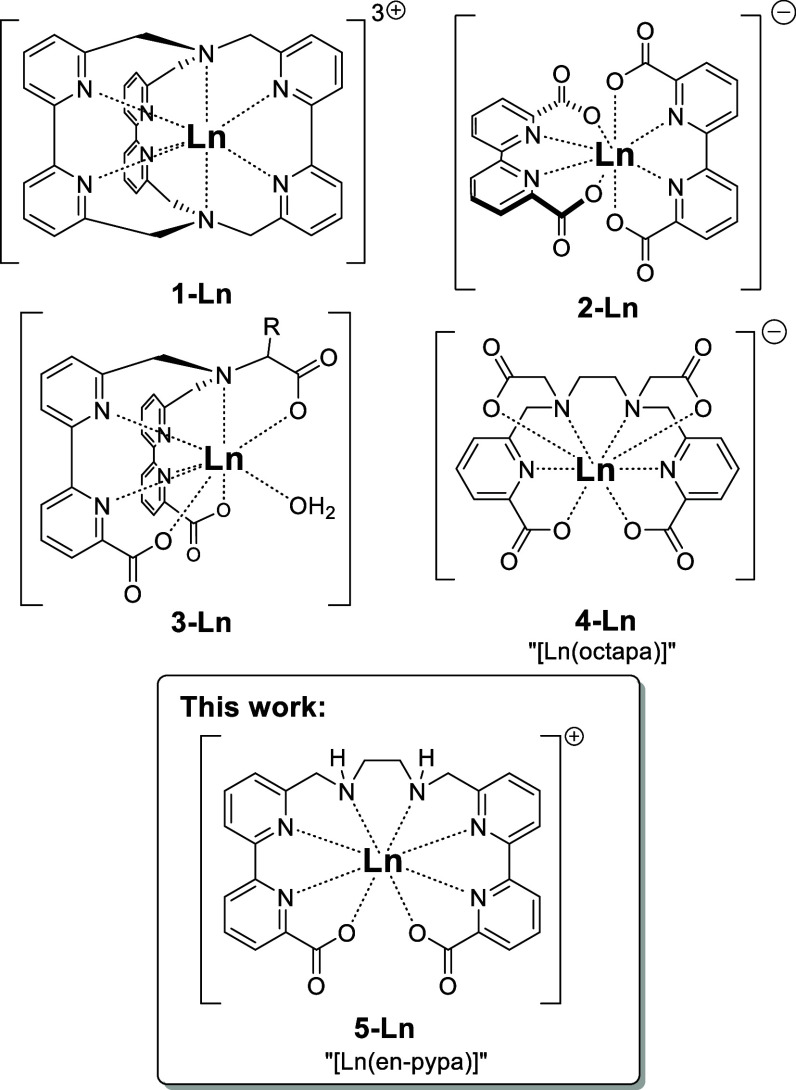
Known metal complexes **1–4** and lanthanoid complexes **5-Ln** with the new ligand “H_2_en-pypa”.

Many of the lanthanoid complexes discussed above suffer from various drawbacks such as insufficient stability under challenging conditions (e.g., **2-Ln**) or very slow complexation kinetics (e.g., **1-Ln**). In this context, Platas-Iglesias et al. were the first to introduce the lanthanoid complexes **4-Ln** with the octadentate chelator “H_4_octapa” (Figure 1)^6a^ which has later also been popularized by Orvig and co-workers for the complexation of lanthanoid radioisotopes.^6b^

In this study, we incorporated various beneficial aspects of ligand design and antennae function for lanthanoid luminescence seen in the previously reported chelator platforms (vide supra) into the new ligand “**en-pypa**” (Figure 1) which gets its name from “ethylene diamine (**en**) **py**ridine **p**icolinic **a**cid”. We report the straightforward synthesis of the ligand as well as its lanthanoid complexes and evaluate the photophysical properties of these new luminophores in aqueous solution.

## Results and Discussion

### Ligand Design – General Strategy

The new ligand H_2_-**en-pypa** (Figure 2) has a number of advantageous design features that make it an attractive scaffold with the possibility of flexible customization, depending on the intended application. From a synthetic perspective, the assembly is especially simple by the modular combination of two main building blocks. The first, the central 1,2-diaminoethane unit, is easily available in many different forms, making it possible to selectively impart chirality by the use of enantiopure diamines (cf. CPL applications) or add secondary functionalizations for the covalent attachment to biomolecules. The second major building block consists of the 2,2′-bipyridine unit, which is known to be a very good and generally applicable sensitizer for lanthanoid luminescence^2^ and which allows very versatile synthetic modification. The latter is especially useful for the modulation of the antenna properties of the bipyridine as well as for the introduction of additional substituents (e.g., for biological tagging purposes).

**Figure 2:**
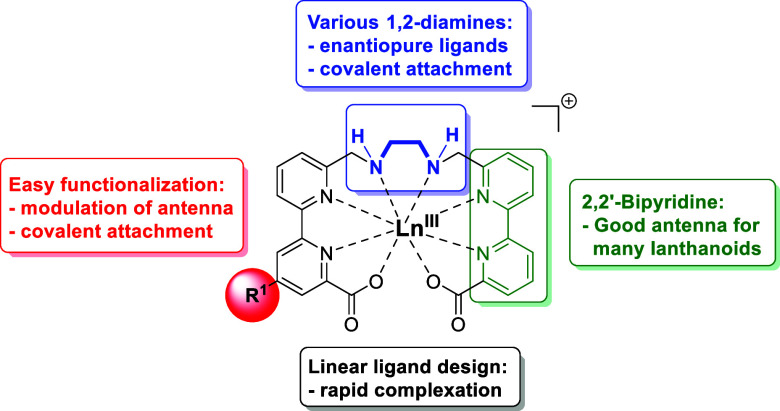
Design features of “**en-pypa**”.

In addition to the criteria mentioned above, the new ligand was also designed to ensure a monocationic nature of the resulting Ln­(III) complexes in order to facilitate future investigations under mass spectrometric conditions in the gas phase.^7^ It should be noted that the presence of the N–H oscillators in **en-pypa** (Figure 2) also has potential drawbacks because of the nonradiative relaxation^8^ that these moieties induce which in turn can diminish luminescence. For this purpose, we also synthesized and analyzed a ligand variation with methylated amines (“H_2_-**Me**_**2**_ **enpypa**”, vide infra).

### Synthesis

The synthesis of H_2_ **-enpypa** (Scheme 1) is straightforward and starts with the methyl diester of 2,2′-bipyridine dicarboxylic acid (**6**)^9^ which is reduced on one side using NaBH_4_ to yield the bipyridine alcohol **7** in low yield (main byproducts are starting material and completely reduced 6,6′-bis­(hydroxymethyl)-2,2′-bipyridine).^10^ Oxidation of the benzylic alcohol group in **7** to the corresponding aldehyde **8** is accomplished with activated manganese dioxide in very good yield. The assembly of the en-pypa scaffold is achieved by 2-fold reductive amination of aldehyde **8** with freshly distilled ethylene diamine under standard conditions using NaBH­(OAc)_3_. Acidic saponification of the ester groups with 6 M hydrochloric acid, followed by preparative RP-HPLC purification (CH_3_CN/H_2_O + 1 vol % CF_3_COOH), gives the trifluoroacetate salt of H_2_ **-enpypa** (**H**_**2**_ **-5**).

**Scheme 1:**
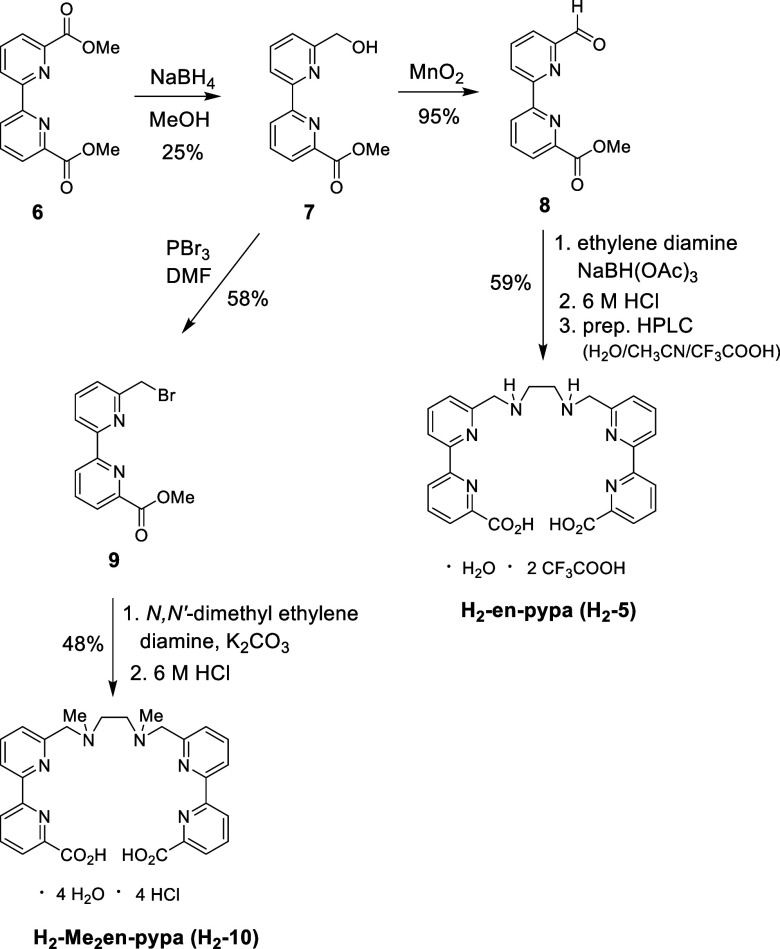
Synthesis of the Ligands H_2_ **-en-pypa** (**H**_**2**_ **-5**) and H_2_ **-Me**_**2**_ **en-pypa** (**H**_**2**_ **-10**)

In order to eliminate the high-energy N–H oscillators in **en-pypa** (Figure 1), the corresponding *N*-methylated ligand H_2_ **-Me**_**2**_ **enpypa** (**H**_**2**_ **-10**) was also a desired modification (Scheme 1). It could not be synthesized using the same route as for **H**_**2**_ **-4** by reductive amination using aldehyde **8**. Instead, the reaction of *N,N*′-dimethyl ethylene diamine with benzylic bromide **9** (prepared by bromination of alcohol **7** with PBr_3_), followed by acidic saponification of the esters gave satisfactory yields of the hydrochloride of H_2_ **-Me**_**2**_ **enpypa** (**H**_**2**_ **-10**) (Scheme 1).

The synthesis of the corresponding lanthanoid complexes with the new ligands (**H**_**2**_ **-5** and **H**_**2**_ **-10**) is very straightforward (Scheme 2). Reaction of the ligands with LnCl_3_·6H_2_O in the presence of NEt_3_ in MeOH is virtually instantaneous (i.e., it only takes a few minutes at most) and yields analytically pure complexes as the chloride salts after precipitation with Et_2_O. Initially, the lanthanoids Sm, Eu, Tb and Dy were chosen for complexation with **H**_**2**_ **-5** due to being the standard emitters in the visible region. In addition, the complex **5-Yb** with the near-IR emitting ytterbium was synthesized. This complex is also suitable for lanthanoid-induced shift analysis in order to extract structural data in solution. This is because the NMR shifts in ytterbium complexes are usually considered to be mostly pseudocontact in nature without strong contact shift contributions. As a diamagnetic and photophysically inactive control system, the complex **5-Lu** was prepared as well. With the *N*-methylated analog on H_2_-Me_2_en-pypa (**H**_**2**_ **-10**) only the ytterbium and lutetium complexes (**10-Yb**/**10-Lu**) were realized.

**Scheme 2:**
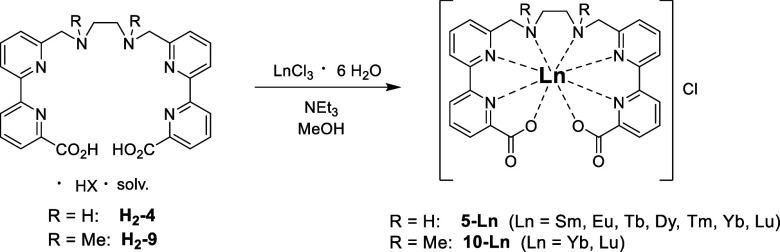
Synthesis of the Complexes **5-Ln** and **10-Ln**

All lanthanoid complexes **5-Ln** and **10-Ln** are fine-grained solids. The complexes **5-Ln** are soluble in a wide variety of polar solvents (e.g., H_2_O, MeOH, CH_3_CN), while the methylated analogues **10-Ln** are not very soluble in aqueous solutions but can be dissolved in a variety of less polar media such as MeOH. All complexes can be stored in solid form at room temperature in air for a long time (>one year) without any sign of deterioration.

### Structural Characterization

^1^H NMR was measured for all complexes in CD_3_OD (see the Supporting Information). In addition, since the goal was to test the luminophores ultimately also in aqueous solution, the complexes **5-Lu** and **5-Yb** were measured in D_2_O. All spectra show one single species in solution with half the number of signals in relation to the protons present in the ligands. Figure 3 shows the spectra of the diamagnetic lutetium complexes **5-Lu** in D_2_O and **10-Lu** in CD_3_OD. Both spectra are quite comparable which means a very similar binding mode for the methylated and nonmethylated ligand. The sharp signals in each case indicate the absence of intermediate/slow exchange processes of any kind. In addition, clear geminal coupling (^2^ *J* ≈ 16 Hz) of the benzylic methylene protons can be seen between 4 and 5 ppm. The latter observation is a very strong indication of chirality, ruling out mirror symmetry in the complexes and suggesting helical, *C*_2_ symmetric arrangements of the ligands around lutetium. The chiral complexes would be, however, formed as a racemate in the absence of absolute stereochemical information during the synthesis. Figure 4 shows the ^1^H NMR spectrum of the complex **5-Yb** in D_2_O. Apart from the moderate paramagnetic line broadening, the spectrum again shows a well-defined complex with an apparent 2-fold symmetry like in the case of its diamagnetic control **5-Lu**. In order to rule out symmetric oligonuclear complexes, mass spectrometry and ion mobility spectrometry (IMS) was employed.

**Figure 3:**
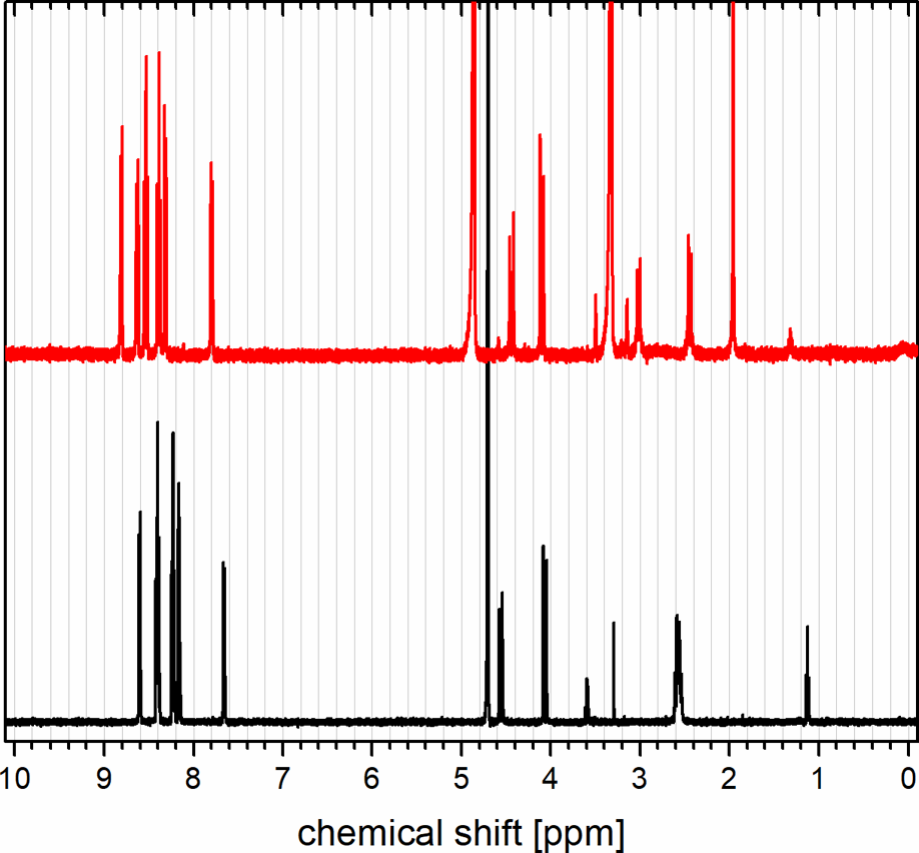
^1^H NMR (400 MHz) spectra of **5-Lu** (D_2_O, black, bottom) and its *N*-methylated analog **10-Lu** (CD_3_OD, red, top).

**Figure 4:**
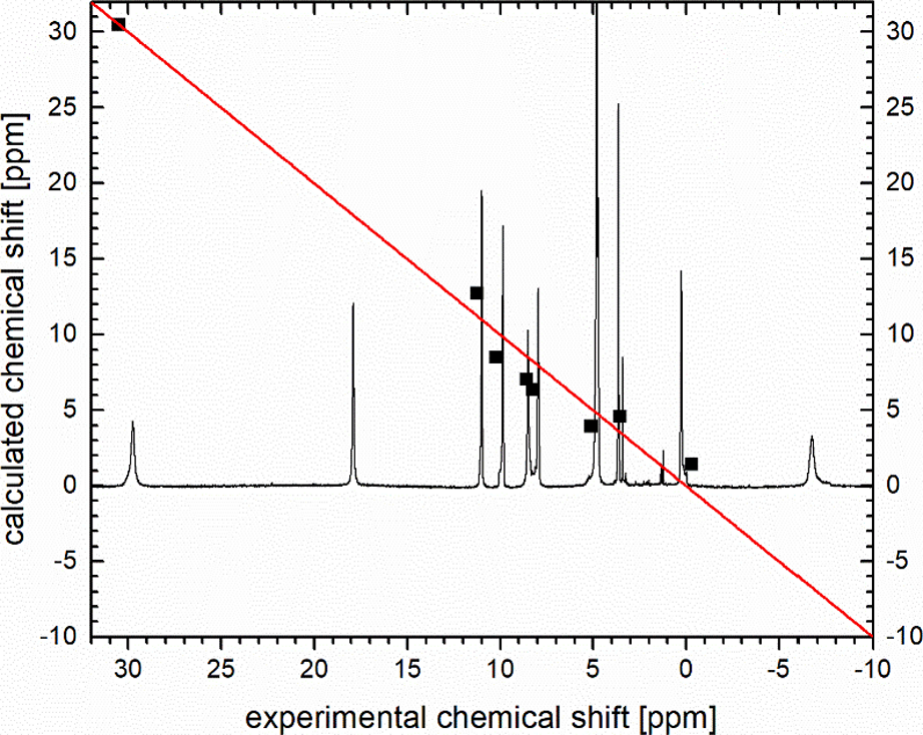
^1^H NMR (400 MHz, D_2_O) spectrum of **5-Yb** and plot of experimental shifts versus those calculated by LIS analysis (see Supporting Information for details). The solid, diagonal line indicates where a perfectly fitting, calculated chemical shift would be situated.

ESI mass spectra of all complexes cleanly show in each case the expected mononuclear species [Ln­(L)]^+^ without indications of higher oligomers. Cyclic IMS (Figure 5) detects only one isomer of the mass corresponding to [Yb­(en-pypa)]^+^ with a calibrated collision cross section (^TW^CCS_N2_) of 208.8 Å^2^. A trajectory method calculation based on the calculated geometry with the IMoS package^11^ gives a theoretical value ^theo^CCS_N2_ of 221.5 Å^2^ which is within 6% of the experimental value and confirms the expected structure of the complex. In conclusion, all data (NMR, MS, IMS) pointed toward the presence of mononuclear species of the form [Ln­(L)]^+^.

**Figure 5:**
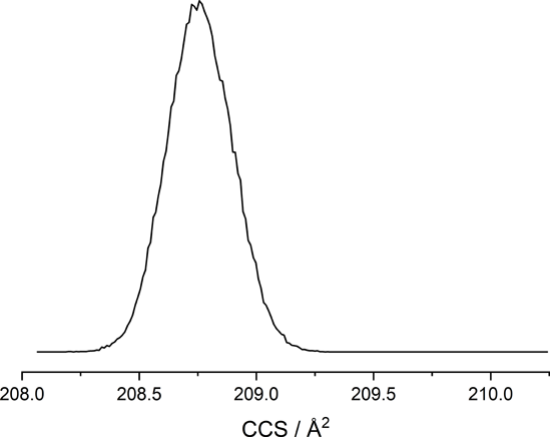
Gas phase mobilogram of [Yb­(en-pypa)]^+^ obtained with cyclic IMS after ESI ionization of **5-Yb** (MeOH).

Unfortunately, extensive attempts to obtain single crystalline material for X-ray analysis were unsuccessful. In order to get further insights into the structure of **5-Yb**, functional theory (DFT) was used. The structure and ground state densities were obtained using the advanced CHYF local hybrid functional^12^ (see Supporting Information for details). The optimized structure of **5-Yb** (Figure 6) shows the expected helically chiral arrangement with almost exact *C*_2_ symmetry.

**Figure 6:**
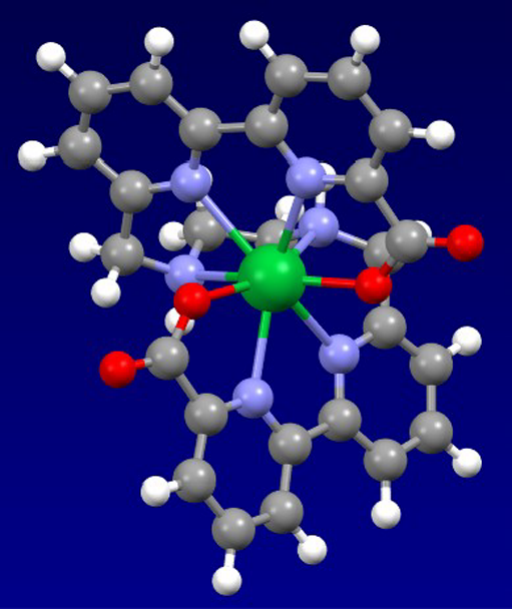
Calculated geometry for one of the enantiomers of racemic **5-Yb**.

The structure also reveals that for the relatively small lanthanoid Yb­(III), there does not seem to be an obvious space for additional ligands (e.g., solvent and especially H_2_O) to bind to the metal. This last assumption is not necessarily also a valid one for the larger lanthanoids and was addressed experimentally through the usual luminescence lifetime measurements in deuterated solvents (vide infra).

Using the calculated structure of **5-Yb**, we performed paramagnetic NMR shift analysis. After initial grouping and partial assignment of protons to shifts and calculated coordinates, an analysis of the lanthanoid induced shifts (LIS) in the ^1^H NMR spectrum of **5-Yb** was performed to complete the assignment and to validate the theoretically derived structural model (see Supporting Information for details).^13^ With an agreement factor of AF = 0.13 a decent fit was found, which is best illustrated by the plot of experimental vs calculated shifts in Figure 4.

### Photophysical Properties

The optical properties of the lanthanoid complexes were investigated in H_2_O and, especially for relatively weakly emitting luminophores, in part also in methanolic solution. UV/vis absorption bands in H_2_O were very similar for all species investigated. Figure 7 shows representative spectra of a few complexes. As expected for 2,2′-bipyridine derivatives, absorption in the UV region is dominated by a band between ca. 290 and 330 nm, usually assigned to mainly nπ*- and ππ*-transitions.

**Figure 7:**
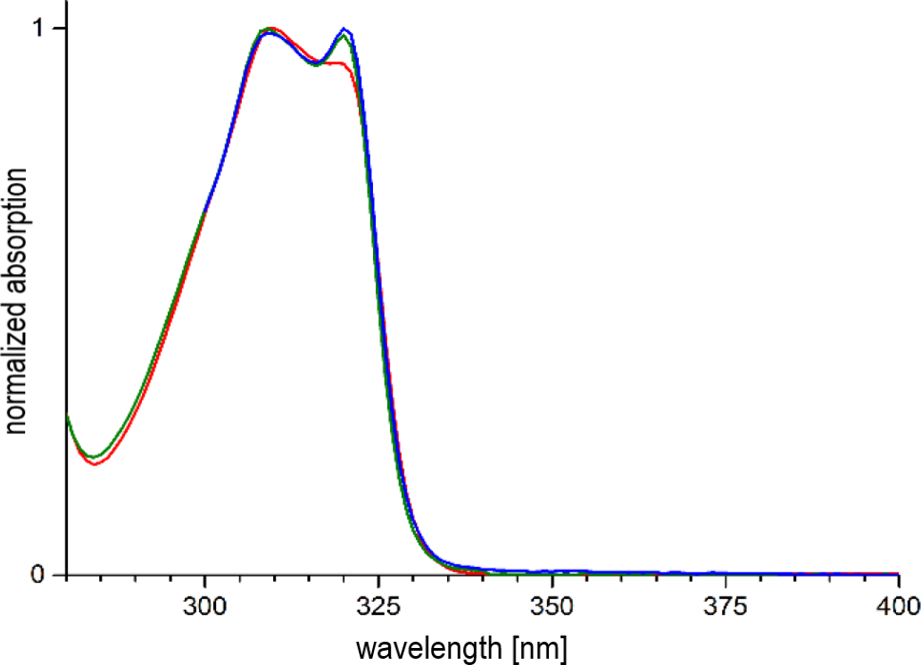
UV/vis absorption spectra of **5-Sm** (red), **5-Dy** (green) and **5-Yb** (blue) in H_2_O.

Next was the determination of the ligand-centered triplet energies T_1_ in the corresponding lutetium complexes **5-Lu** and **10-Lu**. For this purpose, low-temperature (77 K, glass matrix MeOH/EtOH) steady-state phosphorescence spectra of the T_1_ → S_0_ transition were measured (Figure 8).

**Figure 8:**
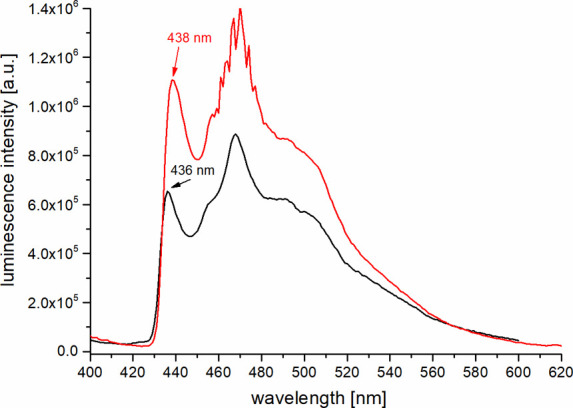
Low temperature emission spectra (λ_exc_ = 310 nm, *T* = 77 K) of the lutetium complexes **5-Lu** (black) and **10-Lu** (red) measured in a MeOH/EtOH glass matrix (1:1, v/v).

The structured phosphorescence bands were very similar for both ligand systems and allowed the determination of the zero-phonon energy *E*(T_1_) ≈ 22,900 cm^–1^. This value is advantageously high in order to act as an antenna for almost all relevant lanthanoids. The criterion for this purpose, *E*(T_1_) – *E*(^2S+1^L_J_) > 2000 cm^–1^,^1^ is fulfilled for most emitting states shown in red in Figure 9. Noteworthy in this context is that even the main emitting levels of Dy (^4^F_9/2_: 21,140 cm^–1^) and Tm (^1^G_4_: 21,370 cm^–1^) are well below *E*(T_1_).^14^ Due to the rather high emitting levels, these two lanthanoids have historically been notoriously hard to sensitize via organic antennae.

**Figure 9:**
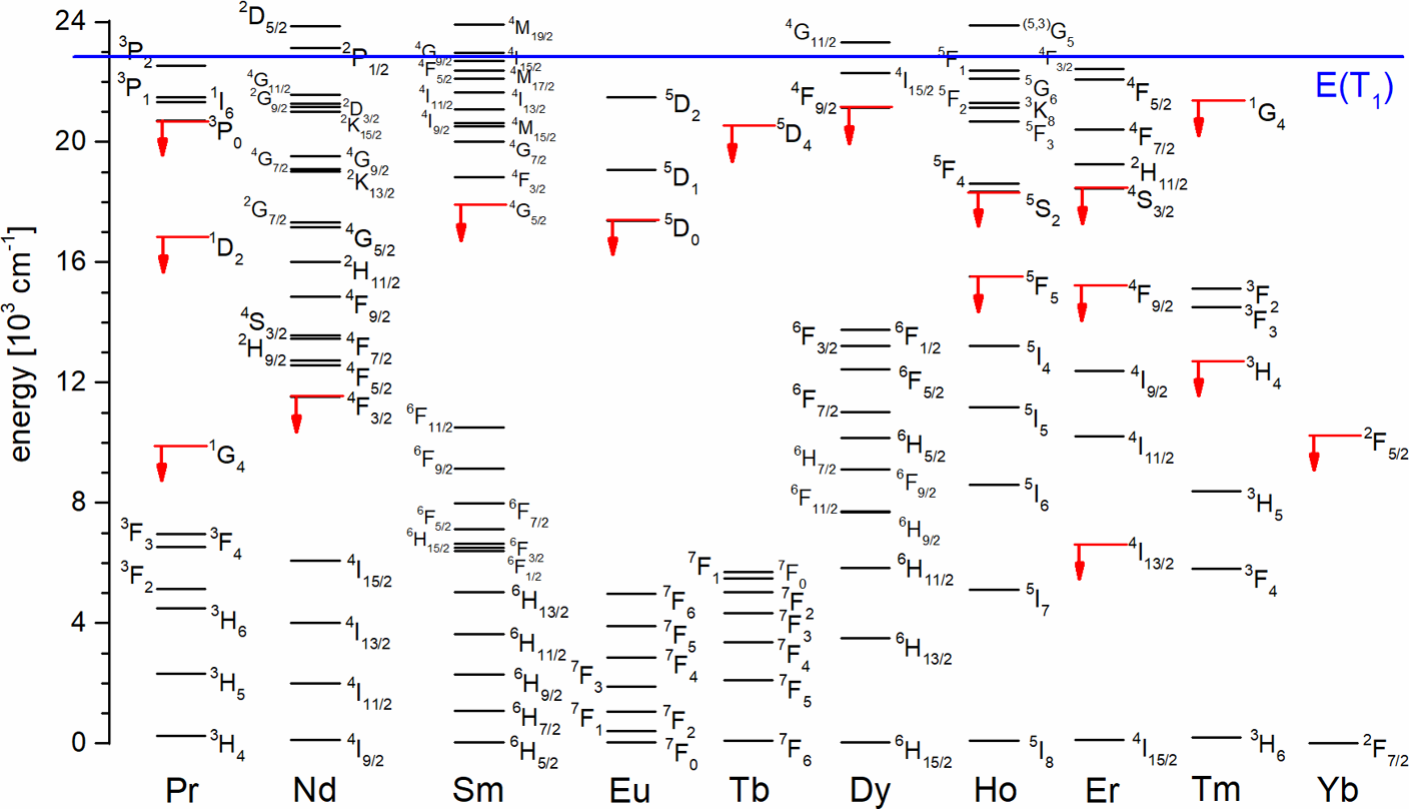
Partial energy level diagram for luminescent trivalent lanthanoids with main emitting lanthanoid levels (red) and triplet energy of the ligands (blue). Adapted with permission from ref 8 (copyright Elsevier B.V. 2018). The term energies are given as calculated for the aqua complexes of Sm–Tm and doped into in LaF_3_ for Yb.^14^

The steady state emission spectra of all complexes in H_2_O showed the emission bands typical for each of the lanthanoids after excitation at λ_exc_ around ca. 310 nm. Figures 10 and 11 show the emission spectra for Sm/Tb and Eu/Dy, respectively.

**Figure 10:**
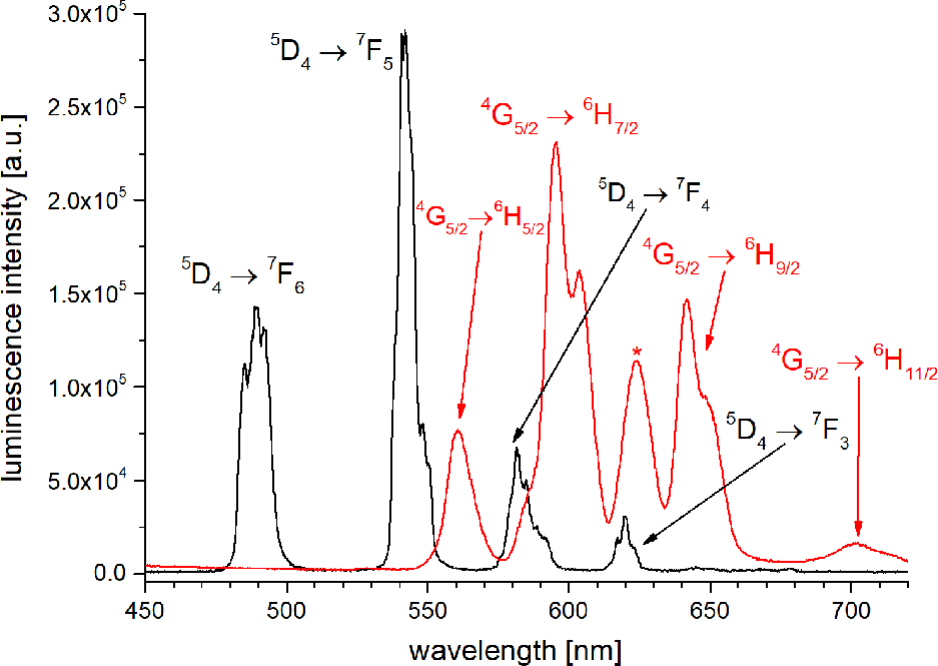
Steady-state emission spectra of **5-Tb** (black, λ_exc_ = 308 nm) and **5-Sm** (red, λ_exc_ = 312 nm, * second order excitation peak) in H_2_O (*c* ≈ 10 μM).

**Figure 11:**
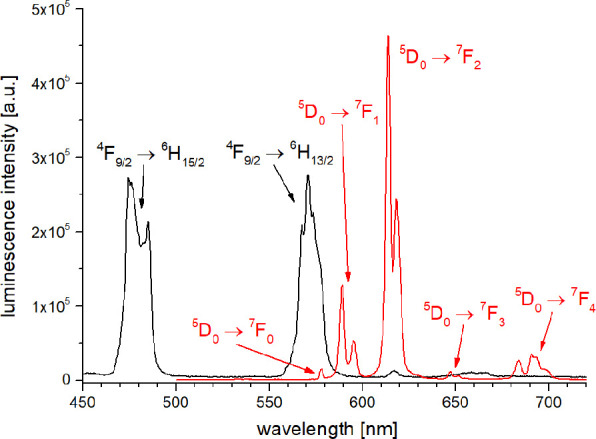
Steady-state emission spectra of **5-Dy** (black, λ_exc_ = 308 nm) and **5-Eu** (red, λ_exc_ = 312 nm,) in H_2_O (*c* ≈ 10 μM).

Surprisingly, even **5-Tm** shows a clear lanthanoid-centered emission band (^1^G_4_ → ^3^H_6_) in the blue region (ca. 460–490 nm) in H_2_O (Figure 12). The emission spectrum also shows strong residual singlet emission around 350 nm, most likely indicating either insufficient intersystem crossing between ligand S_1_ and T_1_ and/or back energy transfer from the ^1^G_4_ level on Tm to the ligand. The observation of Tm luminescence is extremely rare in molecular complexes in solution and only very few examples have been recorded in the past.^19^ As another sign that the new ligands are indeed good sensitizers, the near-IR luminescence in **5-Yb** could also be observed easily in H_2_O (Figure 13).

**Figure 12:**
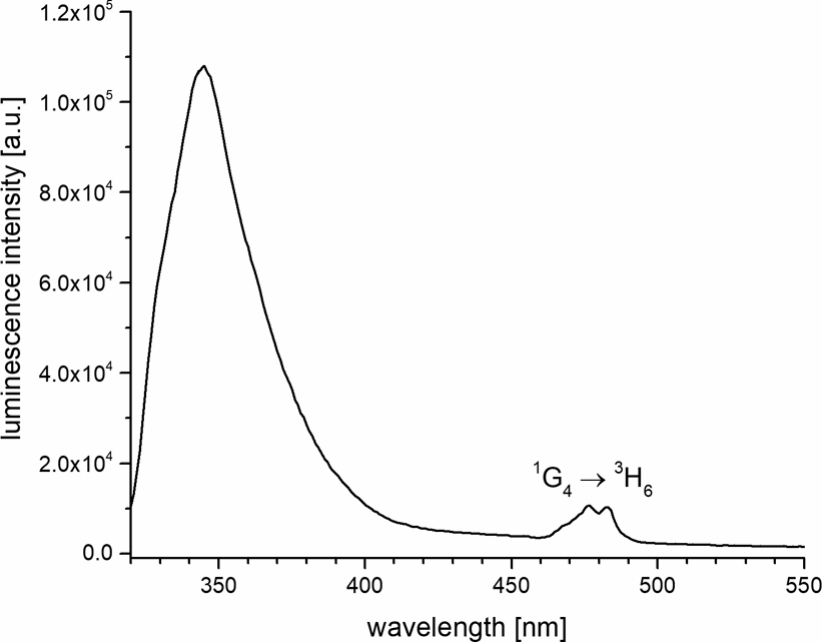
Steady-state emission spectra of **5-Tm** in H_2_O (*c* ≈ 10 μM, λ_exc_ = 310 nm).

**Figure 13:**
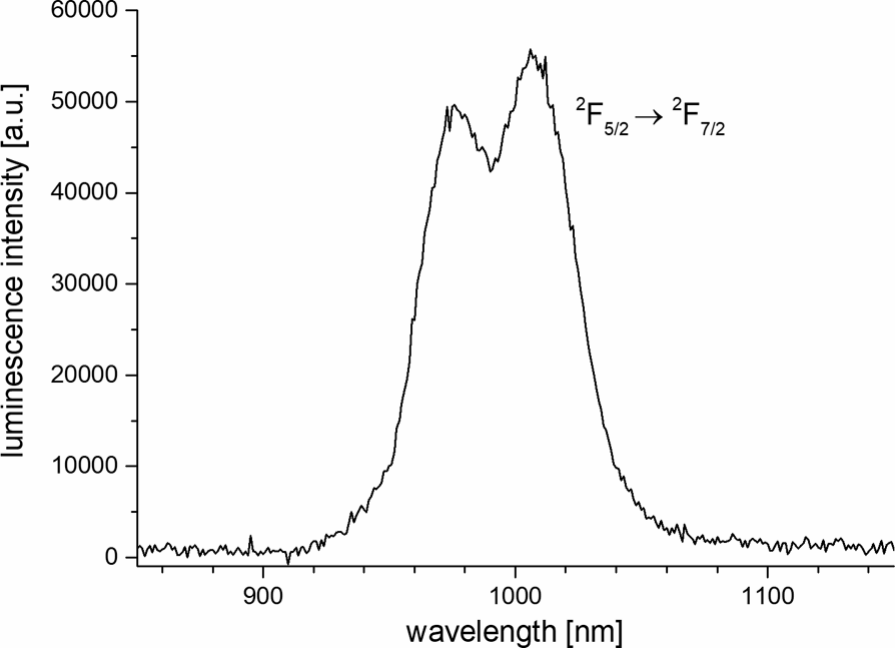
Steady-state emission spectra of **5-Yb** in H_2_O (*c* ≈ 10 μM, λ_exc_ = 309 nm).

Luminescence lifetimes τ_obs_ were determined in aqueous solution at room temperature. All complexes exhibit monoexponential decay curves in H_2_O and D_2_O after excitation in the UV region (λ_exc_ ≈ 308–312 nm), a good indication that in each case only one species should be present. Thulium luminescence in **5-Tm** was too weak and short-lived to acquire reliable lifetime data. During initial experiments, it was observed that the H/D exchange at the secondary amines in the en-pypa complexes (**5-Ln**) was slow but noticeable. For example, stirring the complexes in CD_3_OD led to complete H/D exchange only after 24 h. For the lifetime determinations in deuterated solvents, it was therefore important to freshly prepare solution and to measure quickly in order to avoid obtaining intractable lifetime data due to the presence of mixtures of isotopologic complexes. Table 1 shows a compilation of the lifetime data. The obtained lifetimes in water range from milliseconds for **5-Tb** (τ_obs_ = 1.22 ms) to submicroseconds for **5-Yb** (τ_obs_ = 0.70 μs). The absolute values are moderate to high and compare well with other successful luminescent lanthanoid complexes in the past.^1,20,21^ Expectedly, all lifetimes increase in D_2_O but the extent is not easy to understand in each case. On one hand, it is expected that some lanthanoids such as Sm and Yb with small energy gaps Δ*E* between the emitting and the next lower level show large improvement upon replacement of O–H by O–D oscillators (here improvement in **5-Sm** from τ_obs_ = 11 μs in H_2_O to τ_obs_ = 32 μs in D_2_O or in **5-Yb** from τ_obs_ = 0.7 μs in H_2_O to τ_obs_ = 14 μs in D_2_O). These lanthanoids should be most affected by quenching through high-energy overtones of O–H oscillators in water molecules.^8^ For the same reason, it is not surprising that **5-Tb** only shows a relatively small increase (from τ_obs_ = 1.22 ms in H_2_O to τ_obs_ = 1.87 ms in D_2_O) due to terbium’s large gap Δ*E*. It is, however, a little peculiar why **5-Eu** with a relatively large gap Δ*E* exhibits a rather large improvement (from τ_obs_ = 315 μs in H_2_O to τ_obs_ = 905 μs in D_2_O) while **5-Dy** with dysprosium’s small Δ*E* only shows a moderate increase (from τ_obs_ = 6.3 μs in H_2_O to τ_obs_ = 10.4 μs in D_2_O). While the reason for these differences are still unclear, they prompted us to estimate the number *q* of water molecules in the inner coordination sphere around the lanthanoid by the known empirical equations for Eu, Tb, and Yb.^15,16^ While similar equations were also developed for Sm and Dy in the past,^22^ they are unfortunately considered unreliable for the determination of *q*.^1,20^ One aspect that must be considered for all complexes **5-Ln** is the presence of N–H-oscillators in the ligand backbone. If these are not taken into account, *q* should show artificially high values. If, for example, the standard equation for terbium^15^ (i.e., not considering the quenching effect of N–H oscillators bound to the lanthanoid) is applied to **5-Tb** an apparent *q* = 1.1 is obtained. Taking the additional quenching for N–H into account with the published rates of *k*_N–H_ = 0.09 ms^–1^ for each oscillator,^15^ a more realistic value of *q* = 0.21 (Table 1) results. Clearly, even for the relatively “quenching-resistant” lanthanoid terbium, these oscillators cannot be ignored and therefore must be considered in all calculations for all lanthanoids (if possible). The footnotes for Table 1 consequently show all equations used in full detail. For europium, two suitable equations have been developed in the past by Parker and co-workers^15^ and Horrocks.^16^ The two *q*-values obtained are −0.70 and −0.23, respectively. The negative values in both cases indicate that the equations are not perfectly suited for the ligand en-pypa in **5-Eu**, probably owing to the fact that the equations had originally been developed for aminocarboxylate ligands (mainly DOTA, DTPA and related ligand derivatives).^15,16^ Nevertheless, we interpret the circumstance that both values are below zero as a strong indication that **5-Eu** does not contain water in the inner sphere. In the case of **5-Yb**, the empirical knowledge in the literature is not as voluminous in comparison to the well-studied lanthanoids Eu and Tb. There is a basic equation^14^ but it does not offer the possibility to account for the presence of inner-sphere N–H oscillators. Applying it to the case of **5-Yb** gives an apparent *q*-value of 1.2 (Table 1). Like in the case of **5-Tb** discussed above, this is certainly an overestimation. Here, the *N*-methylated analogue **10-Yb** could be used to elucidate this situation. **5-Yb** and **10-Yb** should have very similar structures in solution as judged by the similarity of the ^1^H NMR spectra of the corresponding lutetium complexes **5-Lu** and **10-Lu** (vide supra). Lifetime determination in H_2_O and D_2_O for **10-Yb**, followed by analysis using the empirical equation for Yb yielded a *q*-value of very close to zero (Table 1: *q* = −0.08). This is rather conclusive evidence that the apparent presence of one water molecule for **5-Yb** (Table 1: *q* = 1.2) is better interpreted as the presence of two inner-sphere N–H moieties of the ligand (in a complex with *q* = 0) which have quenching rates almost equal to two O–H oscillators in one bound water molecule. Our analysis suggests that a quenching rate contribution of *k*_N–H_ ≈ 0.58 μs^–1^ per inner-sphere N–H would be appropriate to correct the current empirical equation for ytterbium in aqueous solution.^14^ The new equation for water would therefore have the following form:

$$q=1\;\mu s\times (1/\tau_{H}-1/\tau_{D}-0.2\;\mu s^{-1}-0.58\;\mu s^{-1}\times n_{N-H})$$

**Table 1:** Luminescence Lifetimes τ_obs_, Absolute Quantum Yields Φ_Ln_^L^ in Aqueous Solution and Calculated Number *q* of Apparent Inner-Sphere Water Molecules^a^

	complex	τ_obs_(H_2_O) (μs)	τ_obs_(D_2_O) (μs)	*q*	λ_exc_/λ_em_ (nm)	Φ_Ln_^L^ (%)
1	5-Sm	11	32	–^b^	312/596	0.05^g^
2	5-Eu	315	905	–0.70^c^	308/570	8.0^g^
				–0.23^d^		
3	5-Tb	1.22 × 10^3^	1.87 × 10^3^	0.21^e^	309/540	37^g^
4	5-Dy	6.3	10.4	–^b^	308/570	0.4^g^
5	5-Yb	0.70	14	1.2^f^	309/1010	n.d.
6	10-Yb	4.5	9.5	–0.08^f^	309/985	0.1^h^

a Estimated uncertainties: lifetimes: ±10%; quantum yields for Eu and Tb: ±10%; quantum yields for Sm, Dy, Yb: ±20%.

b No reliable empirical equation available (see text).

c Reference 15: *q* = 1.2 (1/τ_H_ – 1/τ_D_ – 0.25 – 1.2·*n*_N–H_) with lifetimes in ms.

d Reference 16: *q* = 1.11 (1/τ_H_ – 1/τ_D_ – 0.3 – 0.99·*n*_N–H_) with lifetimes in ms.

e Reference 15: *q* = 5 (1/τ_H_ – 1/τ_D_ – 0.06 – 0.09·*n*_N–H_) with lifetimes in ms.

f Reference 15: *q* = 1/τ_H_ – 1/τ_D_ – 0.2 with lifetimes in μs.

g Measured using quinine sulfate (in 0.1 M H_2_SO_4_) as standard (see ref 17).

h Determined using [Yb­(TTA)_3_phen] as standard (see ref 18).

with lifetimes τ in μs and *n*_N–H_ being the number of inner-sphere N–H moieties.

Absolute quantum yields **Φ**_**Ln**_^**L**^ were determined for complexes **5-Ln** in H_2_O (Table 1). For emission in the visible region (Sm, Eu, Tb, Dy) the luminescence standard quinine sulfate was used,^17^ whereas for the near-IR emitting ytterbium the standard [Yb­(TTA)_3_phen] in toluene was chosen.^18^ For the ytterbium complexes, only the methylated analogue **10-Yb** was investigated. Thulium luminescence from **5-Tm** was too weak in water to be measured accurately. The absolute quantum yield **Φ**_**Ln**_^**L**^ is quite high for the terbium complex (**Φ**_**Ln**_^**L**^ = 37%). In contrast, the value for **5-Eu** (**Φ**_**Ln**_^**L**^ = 8%) is moderate in the context of other successful europium luminophores.^1,20,21^ If the comparison is only taking into account molecular complexes with 2,2-bipyridine building blocks, **5-Eu** and **5-Tb** rank in the top tier.^5,23^ The quantum yields for the new complexes for the generally less luminescent lanthanoids Sm, Dy and Yb are much lower in water (Table 1) with **Φ**_**Ln**_^**L**^ = 0.05% (**5-Sm**), **Φ**_**Ln**_^**L**^ = 0.4% (**5-Dy**) and **Φ**_**Ln**_^**L**^ = 0.1% (**10-Yb**). It should be noted, that for Sm and Dy, these quantum yields are only partial values for the visible range, neglecting the transitions in the near-IR range.^1^ These transitions are also responsible for strongly increased nonradiative deactivation in these lanthanoids.^8^ The absolute values for **5-Sm**, **5-Dy**, and **10-Yb** are small but are in the range of other molecular lanthanoid complexes in aqueous solution.^20^ In this context, the efficiencies are quite respectable. In addition, the values obtained are the lower limits of what could be achieved in less quenching solvents.

## Conclusions

In conclusion, we have designed, synthesized and characterized the new antenna moiety “**en-pypa**” based on universally successful 2,2′-bipyridine units. The ligand design is modular and offers many possibilities for modifications, e.g., by using different 1,2-diamines scaffolds (such as the methylated variant seen in “**Me**_**2**_ **en-pypa**” reported here). Complexation of trivalent lanthanoids using the new, linear ligand is extremely fast (<5 min) and yields mononuclear chelates that are soluble in H_2_O and contain no inner-sphere water molecules. With its very beneficial antenna functionality, i.e., sensitization of luminescence by its high-lying triplet level for the majority of the relevant lanthanoids, this new ligand offers a good compromise between extremely fast complexation with moderate/high luminescence efficiencies in challenging solvents such as water. With these advantageous properties, the new ligand system will be a valuable tool for the development of new analytical luminophores, of new chelators for medical radioisotopes or in other areas of molecular lanthanoid coordination chemistry.

## Experimental Section

### General

**C**hemicals were purchased from commercial suppliers and used as received unless stated otherwise. Deuterated solvents had deuterium contents >99.8%D. Solvents were dried by standard procedures (MeOH: Mg/I_2_; THF/CH_3_CN: solvent purification system MBraun SPS-800; CH_2_Cl_2_: CaH_2_) or purchased in dry form (DMF). Air-sensitive reactions were performed under a dry, dioxygen-free atmosphere of Ar using Schlenk technique. Column chromatography was performed with silica gel 60 (Merck KGaA, 0.040–0.063 mm). Analytical thin layer chromatography (TLC) was done on silica gel 60 F254 plates (Merck, coated on aluminum sheets). Electrospray ionization (ESI) mass spectrometry was measured using Bruker Daltonics Esquire 3000plus. NMR spectra were measured on Bruker Avance II+400 (^1^H: 400 MHz, ^13^C: 101 MHz) or Bruker AVII+500 (^1^H: 500 MHz) or Bruker DPX-250 (^1^H: 250 MHz) or Bruker DPX-200 (^13^C: 50.3 MHz). Chemical shifts (δ) are reported in ppm relative to TMS and the residual solvent signals were used as internal reference.

### HPLC

RP-HPLC was performed using Lichrospher RP-18e columns (Merck, 250 mm × 10 mm – 10 μm) on a Knauer AZURA P6.1L system. Flow rates of 3.0 mL min^–1^ were used. The substances were detected by UV absorption at 300 nm. Mobile phases A: H_2_O (+1% CF_3_COOH, v/v); B: CH_3_CN (HPLC gradient grade).gradient  min%A%B0851558515194555204555358515408515


### Synthesis

#### Bipyridine Alcohol **7**

Under Ar, 2,2′-bipyridine-6,6′-dicarboxylic acid dimethylester (**6**)^9^ (5.74 g, 21.1 mmol, 1.0 equiv) and sodium borohydride (1.92 g, 50.6 mmol, 2.4 equiv) were suspended in dry MeOH (150 mL) and heated under reflux for 3 h. After cooling to room temperature, aqueous HCl (1 M, 50 mL) was added cautiously and the solution was stirred vigorously for 5 min. The pH of the solution was adjusted to ca. 9 using saturated, aqueous Na_2_CO_3_ and the solution was extracted with CH_2_Cl_2_ (3 × 100 mL). The combined organic layers were dried over MgSO_4_ and the solvent was evaporated. The crude product was submitted to column chromatography (SiO_2_, CH_2_Cl_2_/MeOH 25:1). The product was obtained as a colorless solid (1.3 g, 25%).

^1^H NMR (CDCl_3_, 400 MHz): δ = 8.63 (dd, *J* = 7.9 Hz, 1.1 Hz, 1 H), 8.49 (d, *J* = 7.8 Hz, 1 H), 8.15 (dd, *J* = 7.7 Hz, 1.1 Hz, 1 H), 7.99 (t, *J* = 7.8 Hz, 1 H), 7.86 (t, *J* = 7.7 Hz, 1 H), 7.30 (dd, *J* = 7.8 Hz, 1.1 Hz, 1 H), 4.85 (s, 2 H), 4.04 (s, 3 H) ppm. ^13^C NMR (CDCl_3_, 50.3 MHz): δ = 167.7, 158.3, 153.5, 147.9, 139.3, 138.4, 125.7, 124.9, 121.8, 121.3, 100.1, 63.6, 53.1 ppm. MS (ESI, pos. mode): *m*/*z* (%) = 266.8 (100, [M + Na]^+^). TLC: *R*_f_ = 0.36 (SiO_2_, CH_2_Cl_2_/MeOH 9:1, UV-detection). Anal. Calcd for C_13_H_12_N_2_O_3_ (*M*_r_ = 244.25 g/mol): C, 63.93; H, 4.95; N, 11.47; Found: C, 63.87; H, 4.74; N, 11.51.

#### Bipyridine Aldehyde **8**

Bipyridine alcohol **7** (1.15 g, 4.70 mmol, 1.0 equiv) and activated manganese dioxide (2.25 g, 25.9 mmol, 5.5 equiv) were suspended in chloroform (200 mL) and the mixture was heated under reflux for 16 h. After cooling to room temperature, the solution was filtered over Celite and silica gel to remove the remaining manganese dioxide. The solvent was evaporated and the product was obtained as a light-yellow solid (1.08 g, 95%). This material was used for the next steps without further purification.

^1^H NMR (CDCl_3_, 400 MHz): δ = 10.18 (s, 1 H), 8.81 (dd, *J* = 7.0 Hz, 2.0 Hz, 1 H), 8.77 (d, *J* = 7.9 Hz, 1 H), 8.20 (d, *J* = 7.7 Hz, 1 H), 8.06–8.00 (m, 3 H), 4.05 (s, 3 H) ppm. ^13^C NMR (CDCl_3_, 50.3 MHz): δ = 193.5, 165.6, 155.7, 155.3, 152.3, 147.7, 138.2 (2C), 125.7, 125.6, 124.4, 121.9, 52.8 ppm. MS (ESI, pos. mode): *m*/*z* (%) = 294.8 (100, [M+MeOH+Na]^+^), 274.9 (32, [M + Na]^+^). Anal. Calcd for C_13_H_10_N_2_O_3_ (*M*_r_ = 242.23 g/mol): C, 64.46; H, 4.16; N, 11.56; Found: C, 64.23; H, 4.02; N, 11.42.

#### H_2_en-pypa (**H_2_-5**)

Under Ar, bipyridine aldehyde **8** (204 mg, 0.84 mmol, 1.0 equiv) was dissolved in dry CH_2_Cl_2_ (50 mL) and freshly distilled ethylene diamine (28.0 μL, 0.42 mmol, 0.5 equiv) was added. The obtained solution was stirred for 6 h at room temperature. Sodium triacetoxyborohydride (249 mg, 1.18 mmol, 1.4 equiv) was added and the solution was stirred for additional 12 h at room temperature. After the addition of water (20 mL), the solution was adjusted to pH ≈ 9 using saturated, aqueous Na_2_CO_3_. The organic layer was separated and the aqueous layer was extracted with CH_2_Cl_2_ (2 × 20 mL). The combined organic layers were dried over MgSO_4_ and the solvent was evaporated. The residue was dissolved in aqueous HCl (6 M, 40 mL) and heated to reflux for 12 h. After cooling to room temperature, the solvent was removed in vacuo and the residue was dissolved in a minimum of water/CH_3_CN (1:1) and was submitted to preparative HPLC (for conditions see the HPLC section) to yield the product as a colorless solid (167 mg, 59%).

^1^H NMR (250 MHz, 2 M DCl in D_2_O): δ = 8.18 (dd, *J* = 7.3 Hz, 1.5 Hz, 2 H), 8.09 (t, *J* = 7.5 Hz, 2 H), 7.87 (dd, *J* = 7.5 Hz, 1.5 Hz, 2 H), 7.81 (d, *J* = 8.3 Hz, 2 H), 7.63 (t, *J* = 7.9 Hz, 2 H), 7.21­(d, *J* = 7.7 Hz, 2 H), 4.01 (s, 4 H), 3.23 (s, 4 H) ppm. ^13^C NMR (2 M DCl in D_2_O, 50.3 MHz): δ = 161.7, 150.1, 147.5 147.4, 145.1, 141.6, 141.3, 127.4 (2C), 127.0, 123.8, 49.6, 43.0 ppm. ^19^F NMR (235 MHz, 2 M DCl in D_2_O): δ = −76.3 ppm. MS (ESI, pos. mode): *m*/*z* (%) = 484.9 (100, [M + H]^+^). Anal. Calcd for C_26_H_24_N_6_O_4_·H_2_O·2CF_3_COOH (*M*_r_ = 730.58 g/mol): C, 49.32; H, 3.86; N, 11.50; Found: C, 49.65; H, 3.58; N, 11.38.

#### Bipyridine Bromide **9**

Bipyridine alcohol **7** (870 mg, 3.56 mmol, 1.0 equiv) was suspended in DMF (50 mL, peptide grade) and cooled to 0 °C with an ice bath. Phosphorus tribromide (1.00 mL, 10.5 mmol, 3.0 equiv) was added dropwise over a period of ca. 5 min. After complete addition, the obtained solution was stirred at 0 °C for 15 min, allowed to warm to room temperature and stirred for additional 20 h. The solvent was removed in vacuo and saturated, aqueous Na_2_CO_3_ (50 mL) and water (20 mL) were added dropwise cautiously. The obtained solution was extracted with CH_2_Cl_2_ (3 × 100 mL), the combined organic layers were dried over MgSO_4_ and the solvent was evaporated. The crude product was submitted to column chromatography (SiO_2_, CH_2_Cl_2_/MeOH 100:1). The product was obtained as a colorless solid (667 mg, 58%) This compound should be used as soon as possible because it quickly decomposes (within days) in solid substance.

^1^H NMR (CD_2_Cl_2_, 400 MHz): δ = 8.65 (dd, *J* = 7.9 Hz, 1.1 Hz, 1 H), 8.42 (dd, *J* = 7.9 Hz, 0.8 Hz, 1 H), 8.11 (dd, *J* = 7.7 Hz, 1.1 Hz, 1 H), 7.98 (t, *J* = 7.8 Hz, 1 H), 7.87 (t, *J* = 7.8 Hz, 1 H), 7.51 (dd, *J* = 7.7 Hz, 1.0 Hz, 1 H), 4.65 (s, 2 H), 4.00 (s, 3 H) ppm. ^13^C NMR (CD_2_Cl_2_, 101 MHz): δ = 166.2, 157.0, 156.4, 155.6, 148.3, 138.7, 138.5, 125.7, 124.7, 124.4, 121.0, 53.1, 34.7 ppm. MS (ESI, pos. mode): *m*/*z* (%) = 329.0 (100, [M + Na]^+^, Br_1_ pattern), 307.0 (62, [M + H]^+^, Br_1_ pattern), 346.9 (33, [M + K]^+^, Br-pattern). TLC: *R*_f_ = 0.62 (SiO_2_, CH_2_Cl_2_/MeOH 25:1, UV-detection).

#### H_2_Me_2_en-pypa (**H_2_-10**)

Under Ar, bipyridine bromide **9** (200.0 mg, 622.7 μmol, 1.0 equiv) and K_2_CO_3_ (95.0 mg, 685.0 μmol, 1.0 equiv) were dissolved in dry CH_3_CN (20 mL). *N*,*N*′-dimethyl-ethylene diamine (33.5 μL, 311.4 μmol, 0.5 equiv) was added and the obtained suspension was heated to reflux for 20 h. After cooling to room temperature, the suspension was filtered over aluminum oxide and the filter cake was washed with CH_3_CN (150 mL). The filtrate was concentrated, the residue was redissolved in aqueous HCl solution (6 M, 10 mL) and the mixture was heated to reflux for 2 h. After cooling to room temperature, the solvent was removed in vacuo, yielding the product as a light yellow solid (110.2 mg, 48%).

^1^H NMR (2 M DCl in D_2_O, 400 MHz): δ = 7.90–7.81 (m, 4 H), 7.58 (dd, *J* = 7.0 Hz, 2.0 Hz, 2 H), 7.49 (d, *J* = 8.0 Hz, 2 H), 7.31 (t, *J* = 7.9 Hz, 2 H), 6.91 (d, *J* = 7.8 Hz, 2 H), 3.94 (s, 4 H), 3.13 (s, 4 H), 2.15 (s, 6H) ppm. ^13^C NMR (2 M DCl in D_2_O, 100.6 MHz): δ = 160.9, 148.9, 147.2, 145.0, 141.2, 140.4, 127.8, 127.1, 124.1, 123.8, 59.1, 49.8, 41.0 ppm. MS (ESI, pos. mode): *m*/*z* (%) = 513.1 (100, [M + H]^+^). Anal. Calcd for C_28_H_28_N_6_O_4_·4H_2_O·4HCl (*M*_r_ = 730.46 g/mol): C, 46.04; H, 5.52; N, 11.51; Found: C, 46.78; H, 5.24; N, 11.63.

#### Lanthanoid Complexes **5-Ln** and **10-Ln** (General Procedure)

**H**_**2**_ **en-pypa** (**H**_**2**_ **-5**) or **H**_**2**_ **-Me**_**2**_ **en-pypa** (**H**_**2**_ **-10**) (1.0 equiv) and the lanthanoid chloride hexahydrate (1.0 equiv) were suspended in MeOH (1.0 mL per 2.0 μmol Ln) and NEt_3_ (12.0 equiv) was added. The obtained solution was stirred for 10 min at room temperature. The solvent was evaporated and the residue was dissolved in a minimum amount of MeOH. The solution was layered with Et_2_O and stored for 24 h at 4 °C for precipitation. The precipitate was filtered over a Nylon membrane filter (pore size: 0.45 μm), washed with a small amount of ice-cold Et_2_O and dried in vacuo.

##### 5-Sm

From **H**_**2**_ **-5** (30.0 mg, 41.1 μmol), SmCl_3_·6H_2_O (15.0 mg, 41.1 μmol), NEt_3_ (68.3 μL, 492.7 μmol). Yield: 20.3 mg, light yellow solid (78%).

^1^H NMR (CD_3_OD, 400 MHz): δ = 9.13 (br s, 2 H), 8.51 (s br, 4 H), 8.04 (d br, *J* = 7.7 Hz, 4 H), 7.61 (t, *J* = 7.7 Hz, 2 H), 7.46 (d, *J* = 7.4 Hz, 2 H), 7.21 (d, *J* = 7.6 Hz, 2 H), 3.70 (s, 1 H), 3.64 (s, 1 H), 0.15 (s, 2 H), −0.23 (br s, 2 H) ppm. MS (ESI, pos. mode): *m*/*z* (%) = 633.8 (100, [M]^+^, Sm_1_ pattern).

##### 5-Eu

From **H**_**2**_ **-5** (25.0 mg, 35.9 μmol), EuCl_3_·6H_2_O (13.1 mg, 35.9 μmol), NEt_3_ (60.0 μL, 430.8 μmol). Yield: 12.1 mg, colorless solid (53%).

^1^H NMR (CD_3_OD, 400 MHz): δ = 19.3, 14.7, 8.95, 8.21, 7.37, 6.51, 6.46, 5.93, 1.89, −6.40, −12.8 ppm. MS (ESI, pos. mode): *m*/*z* (%) = 635.0 (100, [M]^+^, Eu_1_ pattern).

##### 5-Tb

From **H**_**2**_ **-5** (25.0 mg, 35.9 μmol), TbCl_3_·6H_2_O (13.4 mg, 35.9 μmol), NEt_3_ (60.0 μL, 430.8 μmol). Yield: 17.8 mg, colorless solid (77%).

^1^H NMR (CD_3_OD, 400 MHz): δ = 137.6, 31.7, 6.29, 5.91, 4.50, 1.37, −34.8, −38.6, −49.7, −58.3, −99.8 ppm. MS (ESI, pos. mode): *m*/*z* (%) = 640.8 (100, [M]^+^).

##### 5-Dy

From **H**_**2**_ **-5** (25.0 mg, 35.9 μmol), DyCl_3_·6H_2_O (12.9 mg, 35.9 μmol), NEt_3_ (60.0 μL, 430.8 μmol). Yield: 19.6 mg, light yellow solid (84%).

^1^H NMR (CD_3_OD, 500 MHz): δ = 465.4, 120.3, 21.2, −19.9, −22.2, −45.8, −52.7, −89.2, −247.2, −398.9 ppm. MS (ESI, pos. mode): *m*/*z* (%) = 645.8 (100, [M]^+^, Dy_1_ pattern).

##### 5-Tm

From **H**_**2**_ **-5** (20.0 mg, 27.4 μmol), TmCl_3_·6H_2_O (10.5 mg, 27.4 μmol), NEt_3_ (45.5 μL, 328.5 μmol). Yield: 12.3 mg, colorless solid (69%).

^1^H NMR (CD_3_OD, 400 MHz): δ = 66.2, 50.4, 37.3, 29.9, 27.9, 16.6, −2.85, −9.91, −38.2, −98.1 ppm. MS (ESI, pos. mode): *m*/*z* (%) = 651.0 (100, [M]^+^).

##### 5-Yb

From **H**_**2**_ **-5** (20.0 mg, 27.4 μmol), YbCl_3_·6H_2_O (11.3 mg, 27.4 μmol), NEt_3_ (45.5 μL, 328.5 μmol). Yield: 11.3 mg, colorless solid (52%).

^1^H NMR (CD_3_OD, 200 MHz): δ = 30.5, 18.0, 11.2, 10.2, 8.57, 8.21, 5.08, 3.54, −0.32, −8.46 ppm. MS (ESI, pos. mode): *m*/*z* (%) = 655.7 (100, [M]^+^, Yb_1_ pattern).

##### 5-Lu

From **H**_**2**_ **-5** (10.0 mg, 13.7 μmol), LuCl_3_·6H_2_O (5.3 mg, 13.7 μmol), NEt_3_ (22.8 μL, 164.3 μmol). Yield: 7.6 mg, colorless solid (84%).

^1^H NMR (CD_3_OD, 250 MHz): δ = 8.74 (dd, *J* = 8.0 Hz, 1.0 Hz, 2 H), 8.57 (d, *J* = 7.7 Hz, 2 H), 8.46 (t, *J* = 7.8 Hz, 2 H), 8.31 (t, *J* = 7.6 Hz, 2 H), 8.24 (dd, *J* = 7.5 Hz, 1.5 Hz, 2 H), 7.74 (d, *J* = 7.7 Hz, 2 H), 4.59 (s br, 4 H), 4.12–4.21 (m, 2 H), 2.58–2.74 (m, 4H) ppm. ^13^C NMR (62.9 MHz, CD_3_OD): 172.5, 162.3, 154.9, 153.9, 153.2, 144.3, 143.1, 126.8, 125.6, 125.4, 122.5, 58.1, 53.1 ppm. MS (ESI, pos. mode): *m*/*z* (%) = 656.8 (100, [M]^+^).

##### 10-Yb

From **H**_**2**_ **-10** (15.0 mg, 29.3 μmol), YbCl_3_·6H_2_O (11.3 mg, 29.3 μmol), NEt_3_ (48.7 μL, 351.2 μmol). Yield: 15.9 mg, colorless solid (80%).

^1^H NMR (CD_3_OD, 400 MHz): δ = 35.0, 27.0 (v br), 14.6, 10.5, 4.59, 1.32, −2.26, −3.84 ppm. MS (ESI, pos. mode): *m*/*z* (%) = 684.0 (100, [M]^+^, Yb_1_ pattern).

##### 10-Lu

From **H**_**2**_ **-10** (15.0 mg, 29.3 μmol), LuCl_3_·6H_2_O (11.4 mg, 29.3 μmol), NEt_3_ (48.7 μL, 351.2 μmol). Yield: 16.2 mg, colorless solid (81%).

^1^H NMR (CD_3_OD, 400 MHz): δ = 8.80 (dd, *J* = 8.1 Hz, 0.9 Hz, 2 H), 8.62 (d, *J* = 8.0 Hz, 2 H), 8.52 (t, *J* = 7.9 Hz, 2 H), 8.38 (t, *J* = 7.9 Hz, 2 H), 8.30 (dd, *J* = 7.7 Hz, 0.9 Hz, 2 H), 7.78 (d, *J* = 7.8 Hz, 2 H), 4.42 (d, *J* = 16.1 Hz, 2 H), 4.09 (d, *J* = 16.3 Hz, 2 H), 3.00 (d, *J* = 11.3 Hz, 2 H), 2.41 (d, *J* = 11.2 Hz, 2 H), 1.94 (s, 6H) ppm. MS (ESI+): *m*/*z* (%) = 685.1 (100, [M]^+^).

### Photophysics

UV/vis absorption spectra were recorded on a Jasco-V770 spectrophotometer using 1.0 cm quartz cuvettes. For luminescence measurements, D_2_O and CD_3_OD with deuteration levels of at least 99.8%D and HPLC grade H_2_O were used. Room temperature measurements were performed in quartz cuvettes (Suprasil, 1 cm). Low temperature spectra were recorded on frozen glasses of solutions using a dewar cuvette filled with liquid N_2_ (*T* = 77 K). All experiments were performed on one of the two following instruments.

The first was a Horiba Fluorolog-3 DF equipped with a 450 W xenon lamp for steady state spectra and a pulsed xenon lamp (FWHM of excitation pulse approximately 2 μs) for lifetime measurements. Emission was detected by a Hamamatsu R2658P PMT detector (200 nm < λ_em_ < 1010 nm) in the visible region or by a Hamamatsu H10330-75 PMT detector (950 nm < λ_em_ < 1700 nm) in the near-IR. Spectral selection in the excitation path was accomplished by a DFX monochromator (double gratings: 1200 grooves/mm, 330 nm blaze) and in the emission paths in the visible/near-IR spectral region (λ_em_ < 1010 nm) by a spectrograph iHR550 (single gratings: either 1200 grooves/mm, 500 nm blaze or 950 grooves/mm, 900 nm blaze) and in the near-IR spectral region (λ_em_ > 950 nm) by a spectrograph iHR320 (single grating: 600 grooves/mm, 1000 nm blaze). High resolution spectra of **5-Dy** were measured on this instrument using the standard emission grating (1200 grooves/mm, 500 nm blaze) and 0.1 nm emission bandpass.

The second fluorimeter was a PTI Quantamaster QM4 equipped with a 75 W continuous xenon short arc lamp as excitation source. Emission was monitored using a PTI P1.7R detector module (Hamamatsu PMT R5509-72 with a Hamamatsu C9525 power supply operated at 1500 V and a Hamamatsu liquid N2 cooling unit C9940 set to −80 °C). Spectral selection was achieved by single grating monochromators (excitation: 1200 grooves/mm, 300 nm blaze; near-IR emission: 600 grooves/mm, 1200 nm blaze).

For both instruments, luminescence lifetimes were determined with a xenon flash lamp as excitation source (Hamamatsu L4633: pulse width ca. 1.5 μs FWHM). Lifetime data analysis (deconvolution, statistical parameters, etc.) was performed using the software package FeliX32 from PTI or DAS analysis from Horiba. Lifetimes were either determined by tail-fitting (for very long lifetimes) or by deconvolution of the decay profiles with the instrument response function (IRF). IRFs were measured using a dilute aqueous dispersion of colloidal silica (Ludox AM-30). Absolute quantum yields were determined using the following equation:

$$\Phi_{x}=\Phi_{r}\times ({\mathrm{Grad}}_{x}/{\mathrm{Grad}}_{r})\times (n_{x}^{2}/n_{r}^{2})$$

where *n* is the refractive index (H_2_O: *n* = 1.330; toluene: *n* = 1.496) and Grad is the linearly fitted slope from the plot of the integrated luminescence intensity versus the absorbance at the excitation wavelength. The subscripts ‘x’ and ‘r’ refer to the sample and reference, respectively. For visible emission, quinine sulfate in 0.1 M H_2_SO_4_ was used as a reference material with a fluorescence quantum yield of Φ_r_ = 54.6%.^17^ For the near-IR emitter **5-Yb**, [Yb­(tta)_3_phen] in toluene was used with a quantum yield of =1.1%.^18^

### Ion Mobility Mass Spectrometry

Ion mobility (cyclic-IMS) measurements of collision cross sections in N_2_ (^TW^CCS_N2_) were performed using a Waters Cyclic-IMS instrument following methods and calibration procedures as outlined in ref 24.

## Supplementary Material



## Notes

^1^H NMR spectra of intermediates and the complexes **5-Ln** and **10-Ln**, HRMS spectra for complexes **5-Ln** and **10-Ln,** details for the lanthanoid induced NMR shift analysis for **5-Yb**, and details for the quantum chemical calculations (PDF)
