# Supplementary material for: Lanthanoid Luminophores with Linear, Bipyridine-Based Antenna Ligands
Source: Inorg Chem. 2025 Oct 28;64(44):22048–58. doi: 10.1021/acs.inorgchem.5c03839 (PMC12606711; doi:10.1021/acs.inorgchem.5c03839)
Supplement: Supplementary file 1 [file ic5c03839_si_001.pdf]

Lanthanoid Luminophores with Linear, Bipyridine-Based Antenna Ligands

Supporting Information

Christian Kruck,† Timo Neumann,† Alexander Schäfer,‡ Elisabeth Kreidt,# Patrick Weis,‡ Christof Holzer,§ Manfred M. Kappes*,‡ and Michael Seitz*,†

† Institute of Inorganic Chemistry, University of Tübingen, Auf der Morgenstelle 18, 72076 Tübingen, Germany

‡ Institute of Physical Chemistry, Karlsruhe Institute of Technology (KIT), 76131 Karlsruhe, Germany and Institute of Nanotechnology, Karlsruhe Institute of Technology (KIT), 76344 Eggenstein-Leopoldshafen, Germany

Department of Chemistry and Chemical Biology, Technical University Dortmund, Otto-Hahn-Str. 6, 44227 Dortmund, Germany

§ Institute for Quantum Materials and Technologies, Karlsruhe Institute of Technology (KIT), 76131 Karlsruhe, Germany

Email: michael.seitz@uni-tuebingen.de ; manfred.kappes@kit.edu

Supporting Information

Table of Contents — Page

1	^{1}H NMR Spectra of Intermediates and 5-Ln / 10-Ln	S2
2	Mass Spectra of Complexes 5-Ln / 10-Ln	S8
3	Lanthanoid-Induced NMR Shift Analysis	S14
4	Details for Quantum Chemical Calculations	S16
5	References	S17

1 ^{1}H NMR Spectra of Intermediates and 5-Ln / 10-Ln

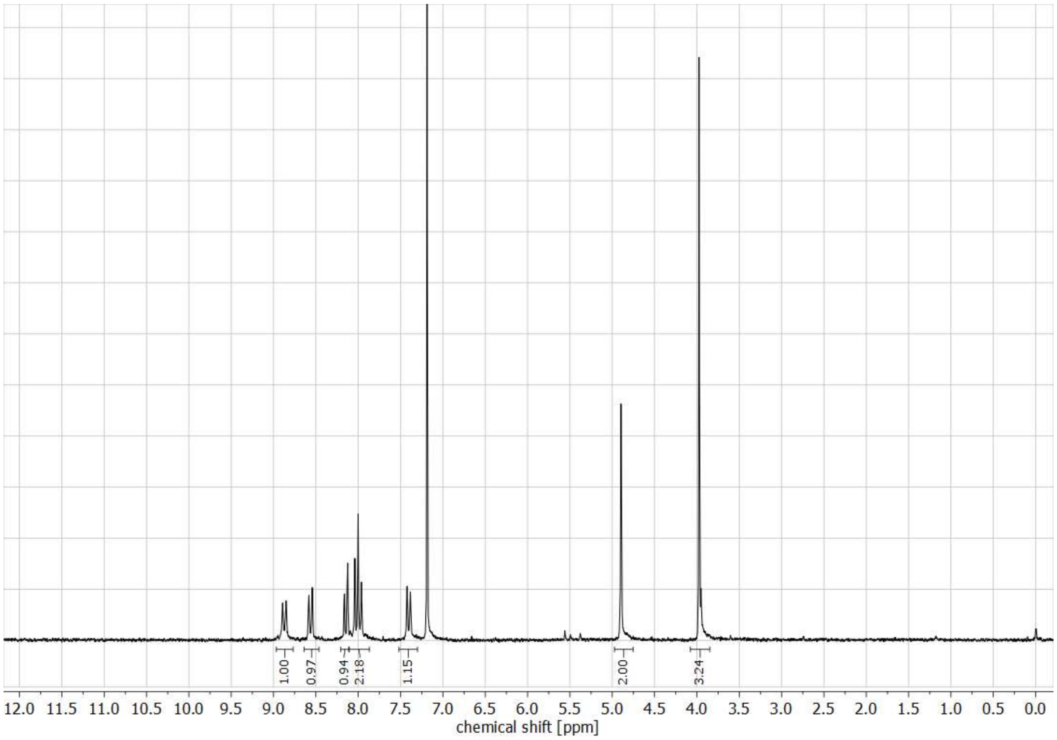

Figure S1. ^{1}H NMR ($CDCl_3$, 400 MHz) spectrum of **7.**

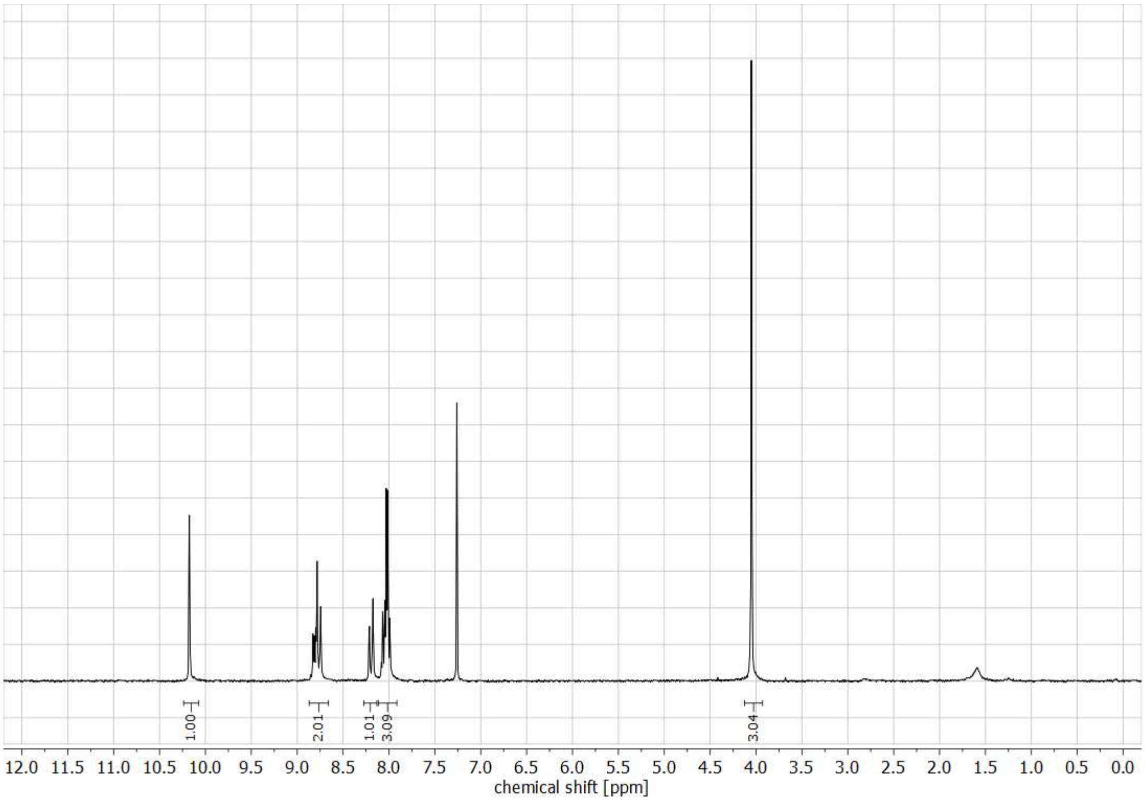

Figure S2. ^{1}H NMR ($CDCl_3$, 400 MHz) spectrum of **8.**

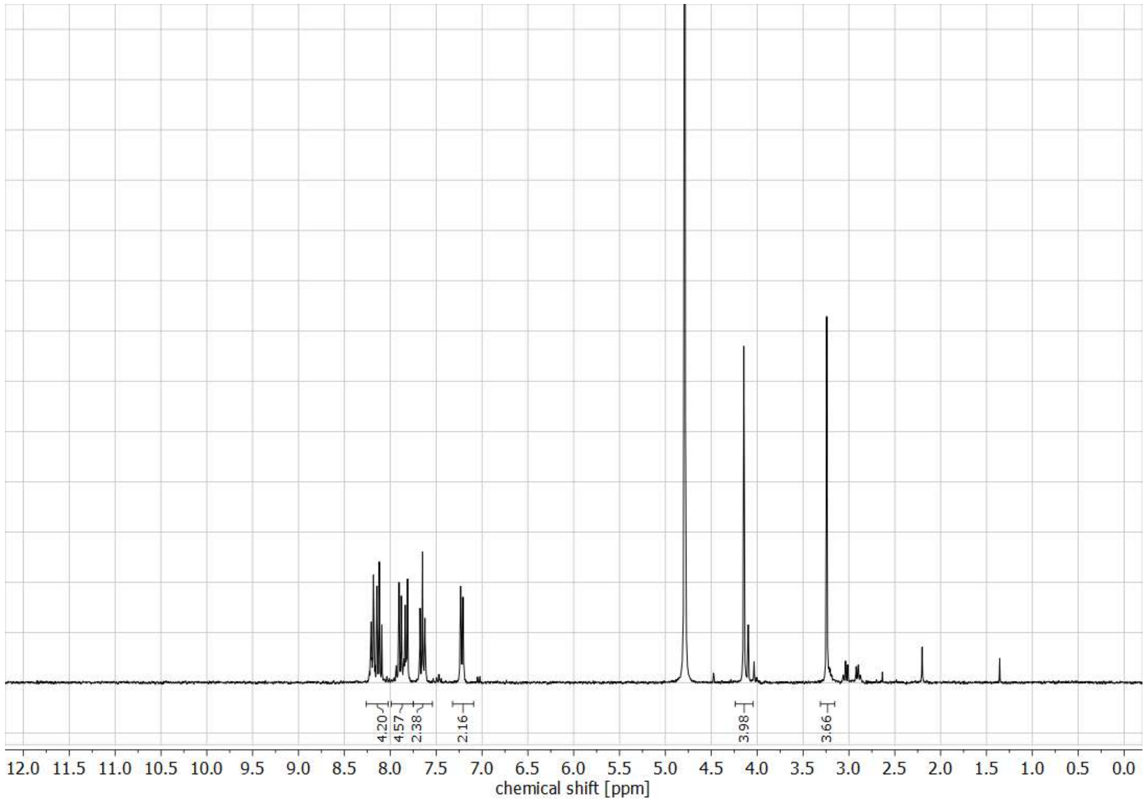

Figure S3. ^{1}H NMR (2 M DCl in D_2O, 300 MHz) spectrum of H_2en-pypa**.**

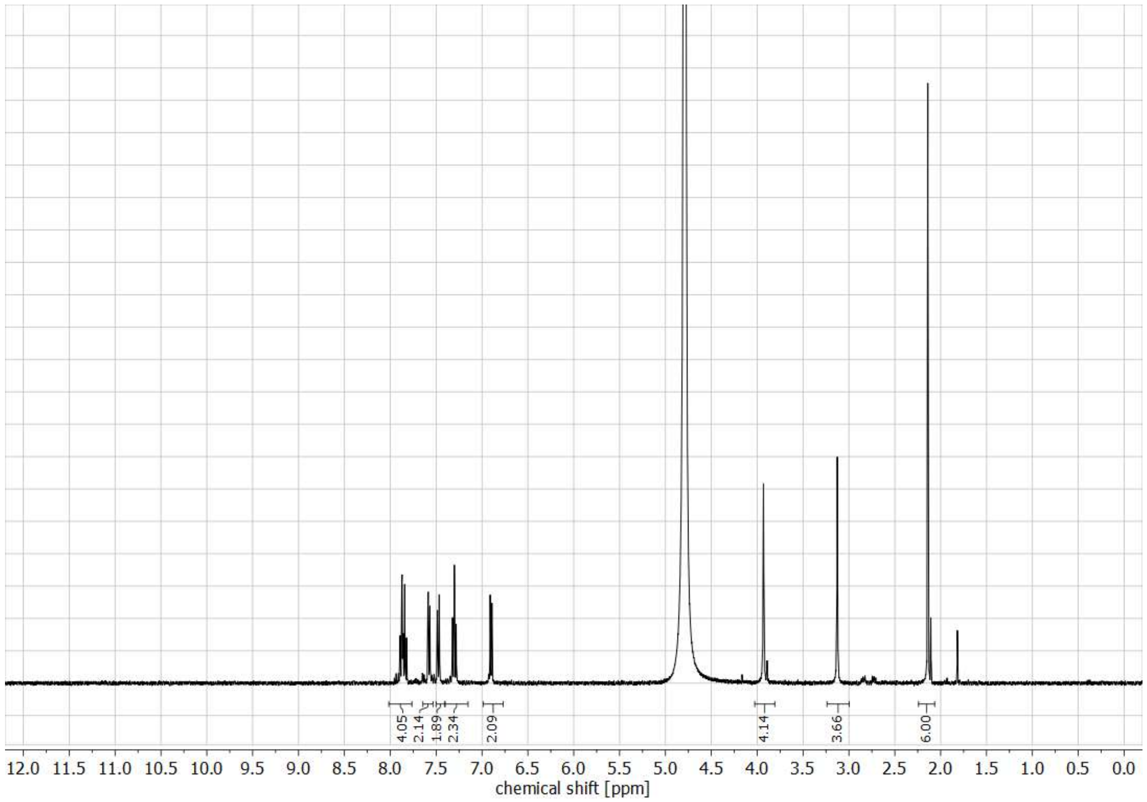

Figure S4. ^{1}H NMR (2 M DCl in D_2O, 400 MHz) spectrum of H_2Me_2en-pypa.

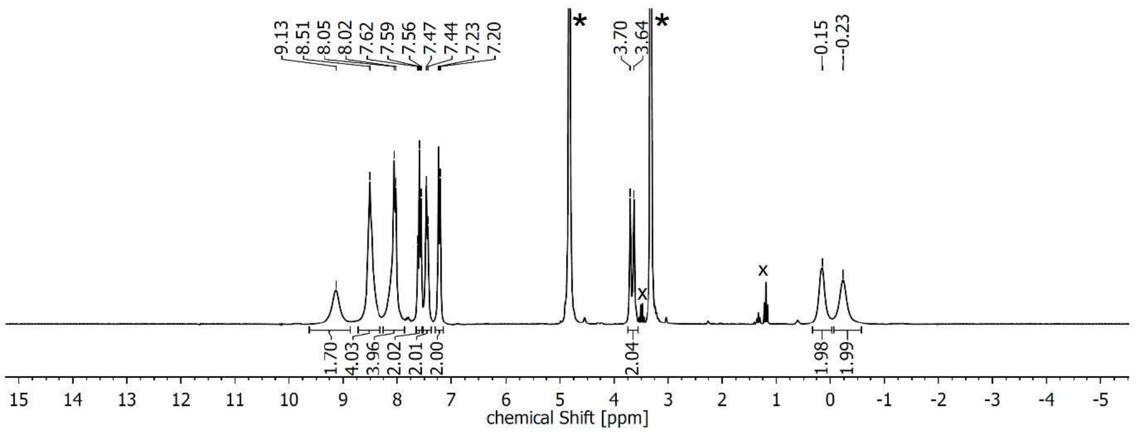

Figure S5. ^{1}H NMR (CD_3OD, 400 MHz) spectrum of **5-Sm** (* and x denote residual solvent and traces of Et_2O, respectively).

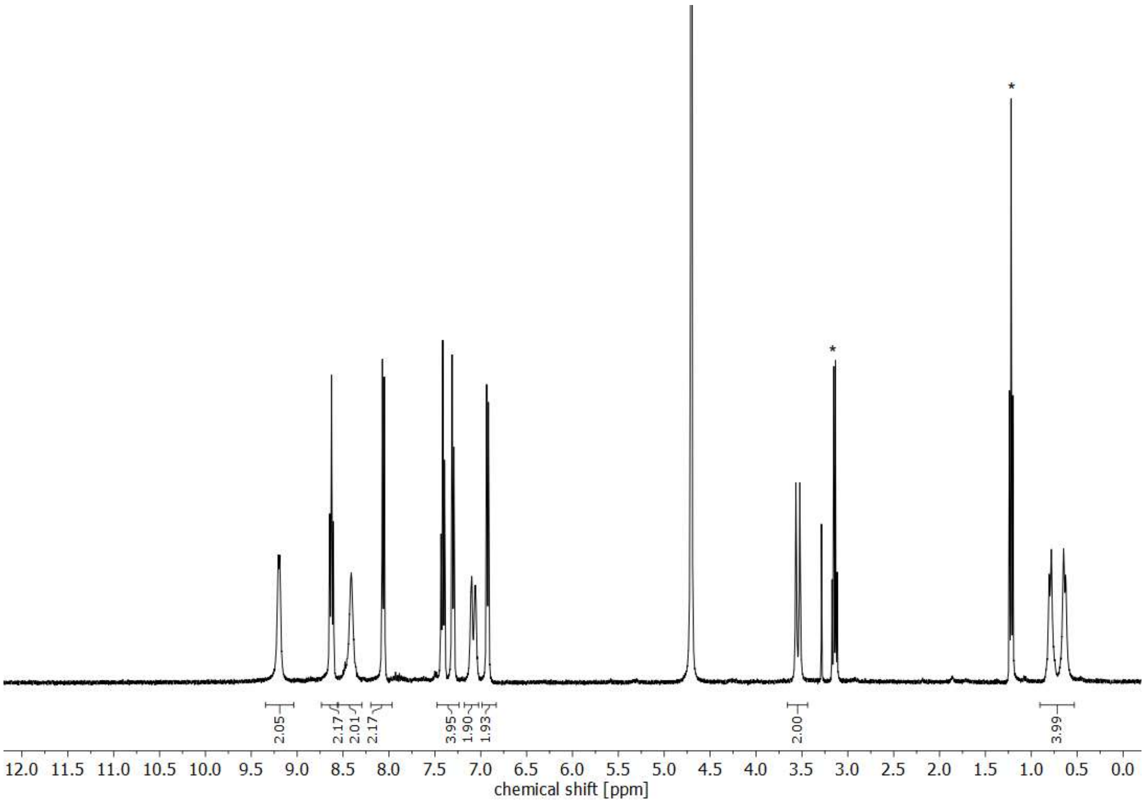

Figure S6. ^{1}H NMR (D_2O, 400 MHz) spectrum of **5-Sm** (*denotes residual Et_2O).

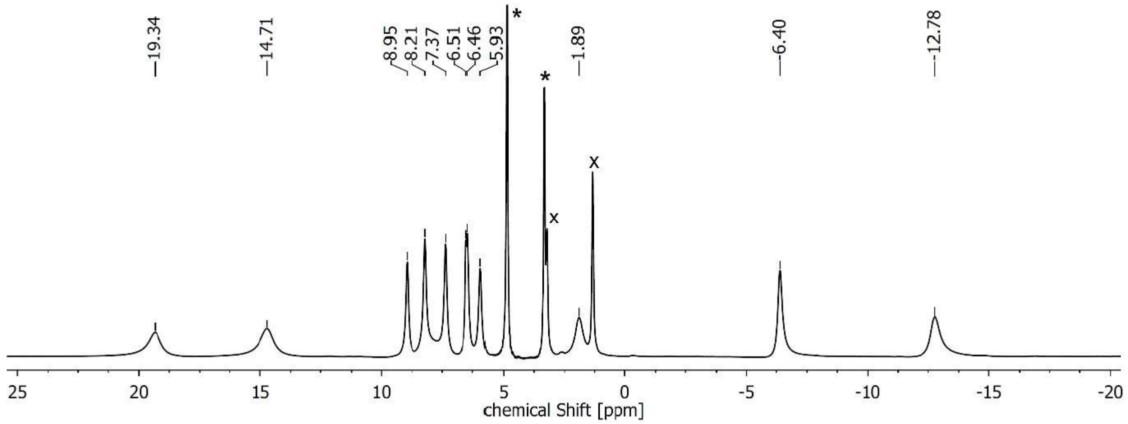

Figure S7. ^{1}H NMR (CD_3OD, 400 MHz) spectrum of **5-Eu** (* and x denote residual solvent and traces of Et_2O, respectively).

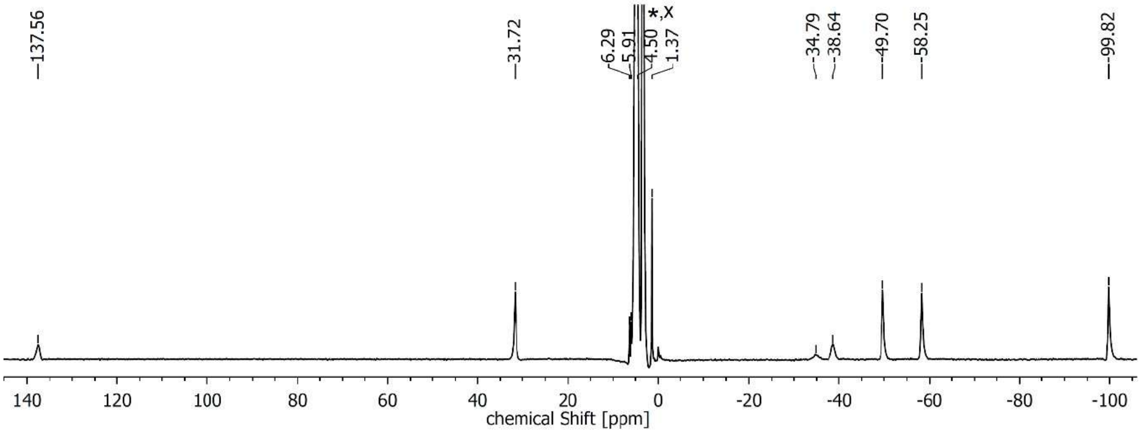

Figure S8. ^{1}H NMR (CD_3OD, 400 MHz) spectrum of **5-Tb** (* and x denote residual solvent and traces of Et_2O, respectively).

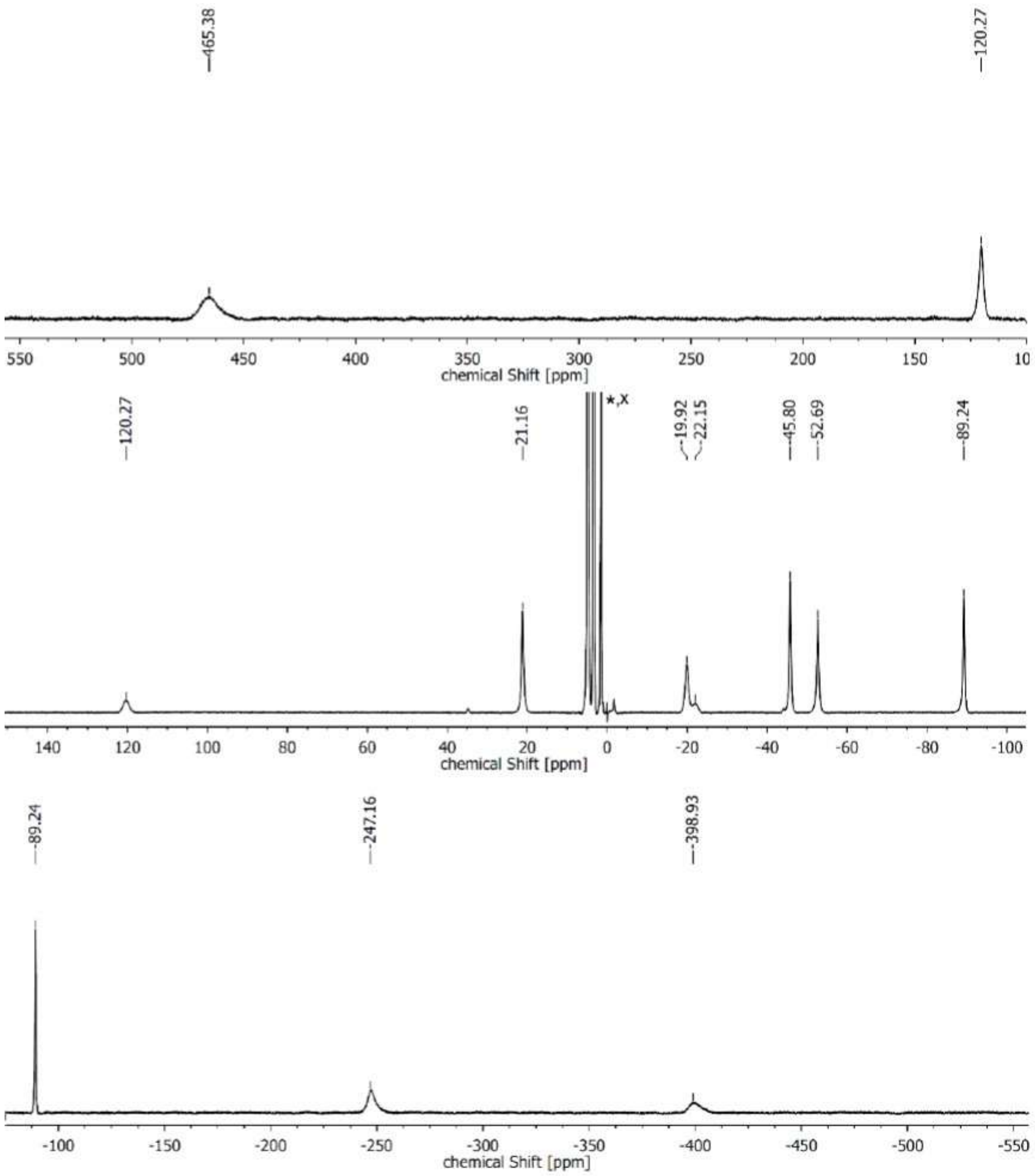

Figure S9. ^{1}H NMR (CD_3OD, 500 MHz) spectrum of **5-Dy** (* and x denote residual solvent and traces of Et_2O, respectively).

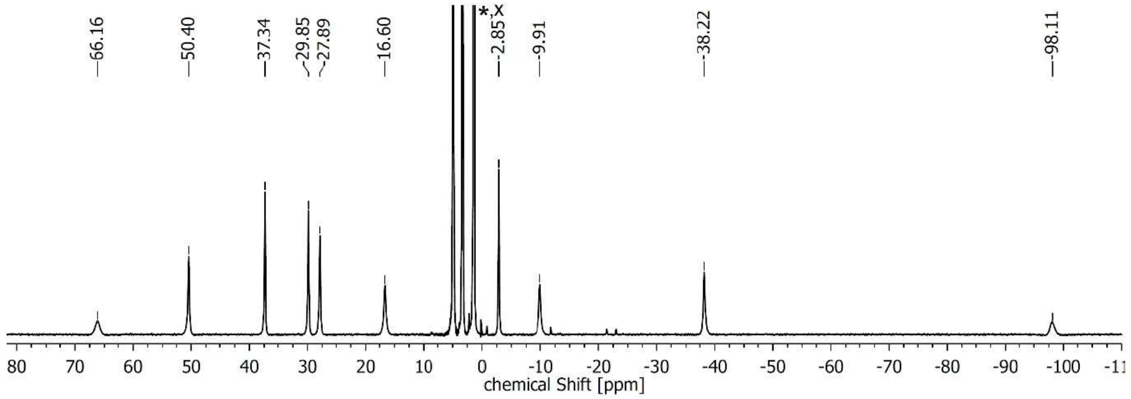

Figure S10. ^{1}H NMR (CD_3OD, 400 MHz) spectrum of **5-Tm** (* and x denote residual solvent and traces of Et_2O, respectively).

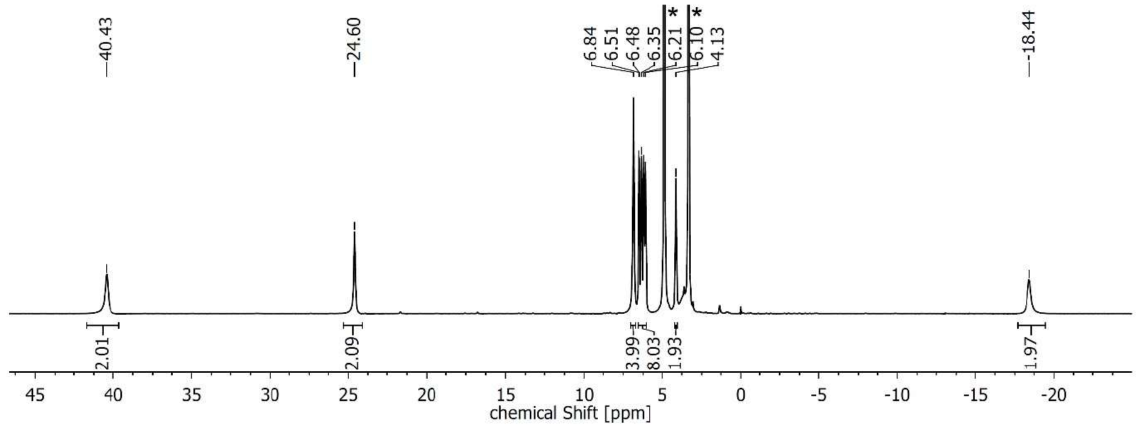

Figure S11. ^{1}H NMR (CD_3OD, 400 MHz) spectrum of **5-Yb**.

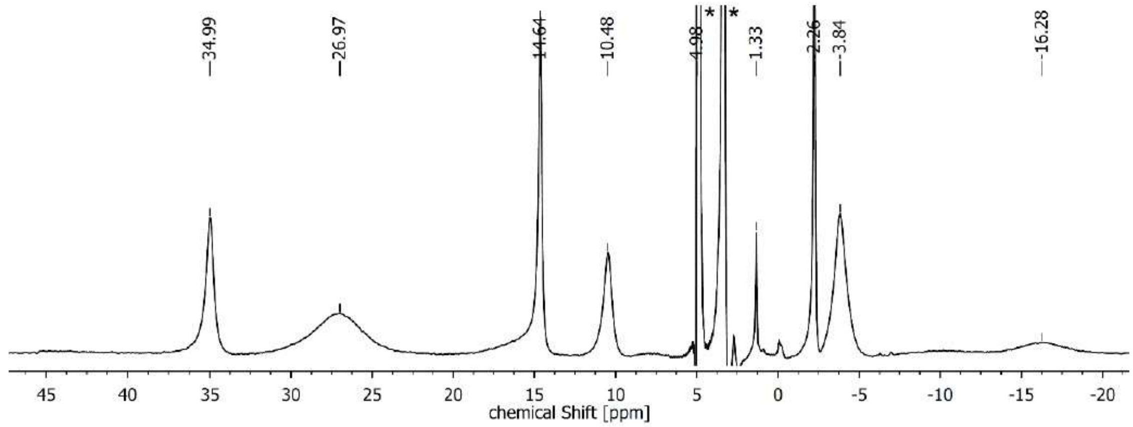

Figure S12. ^{1}H NMR (CD_3OD, 400 MHz) spectrum of **10-Yb**.

2 Mass Spectra of Complexes 5-Ln / 10-Ln

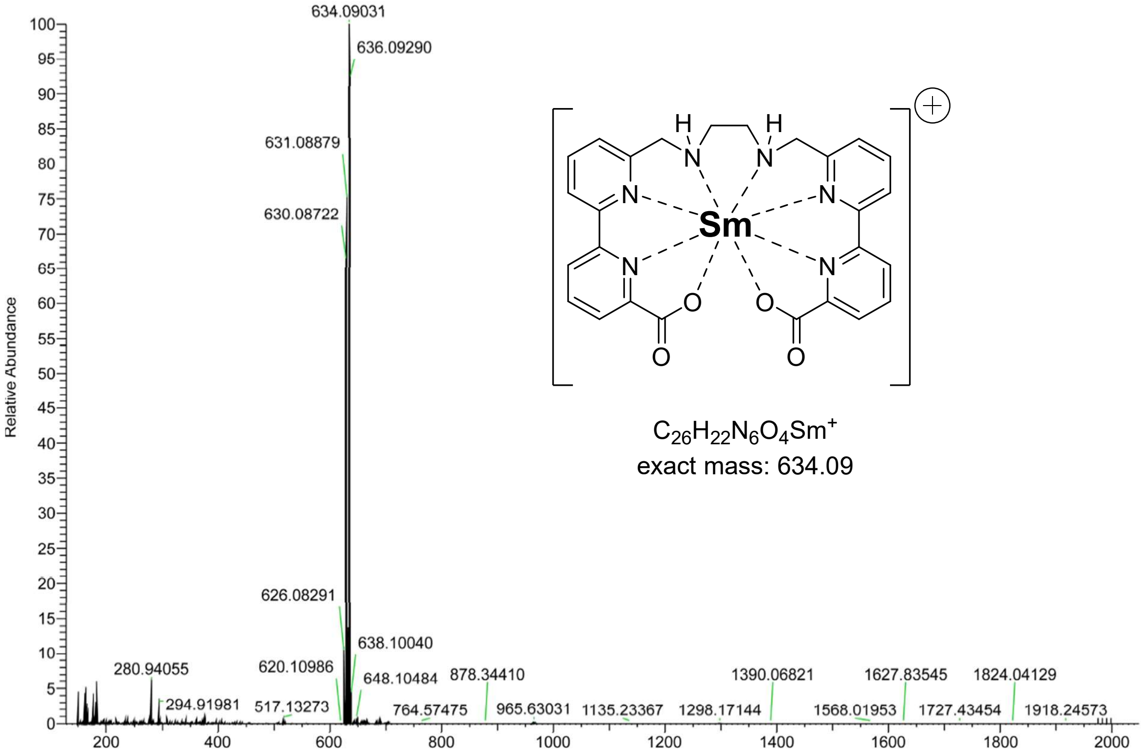

Figure S13. High resolution mass spectrum (ESI, pos. mode) of **5-Sm**.

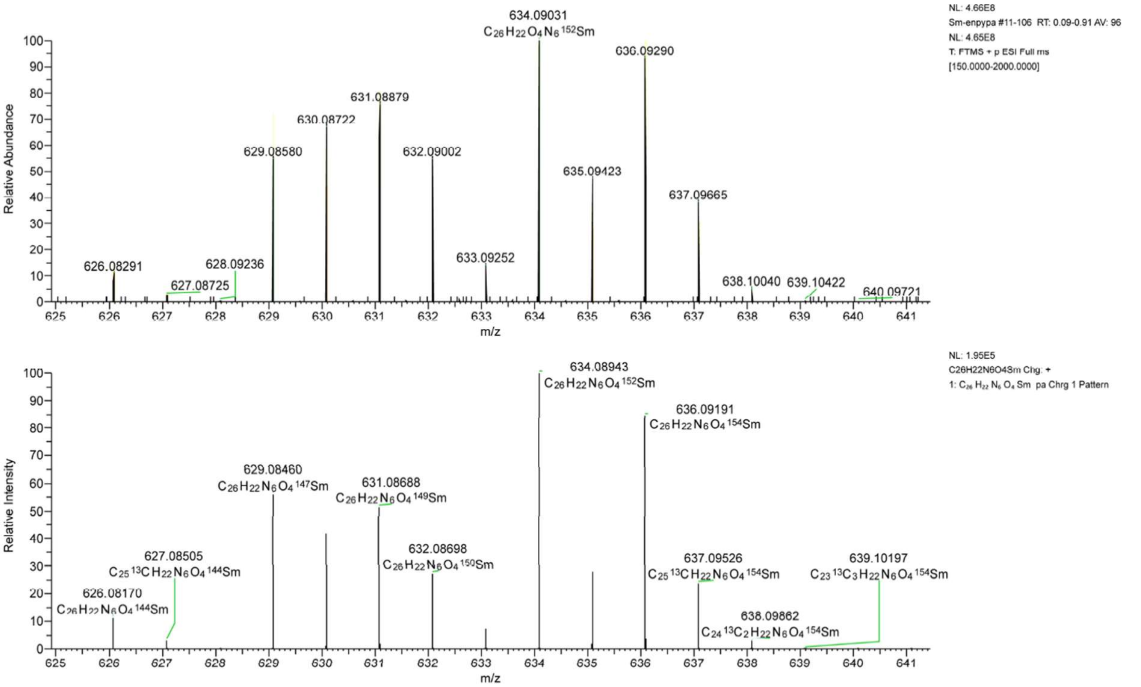

Peak Mass	Display Formula	S Fit	RDB	Delta [ppm]	Theo. mass	Rank	Combined Score	MS Cov. [%]	Pattern Cov. [%]	MSMS Matched...
634.09031	$C_{26}H_{22}O_4N_6{}^{152}Sm$	5.99999684406545	19.00	1.38	634.08943	1	41.91	43.91	52.01	(Collection)

Figure S14. Measured (top) vs. simulated (bottom) molecular ion peak of **5-Sm**.

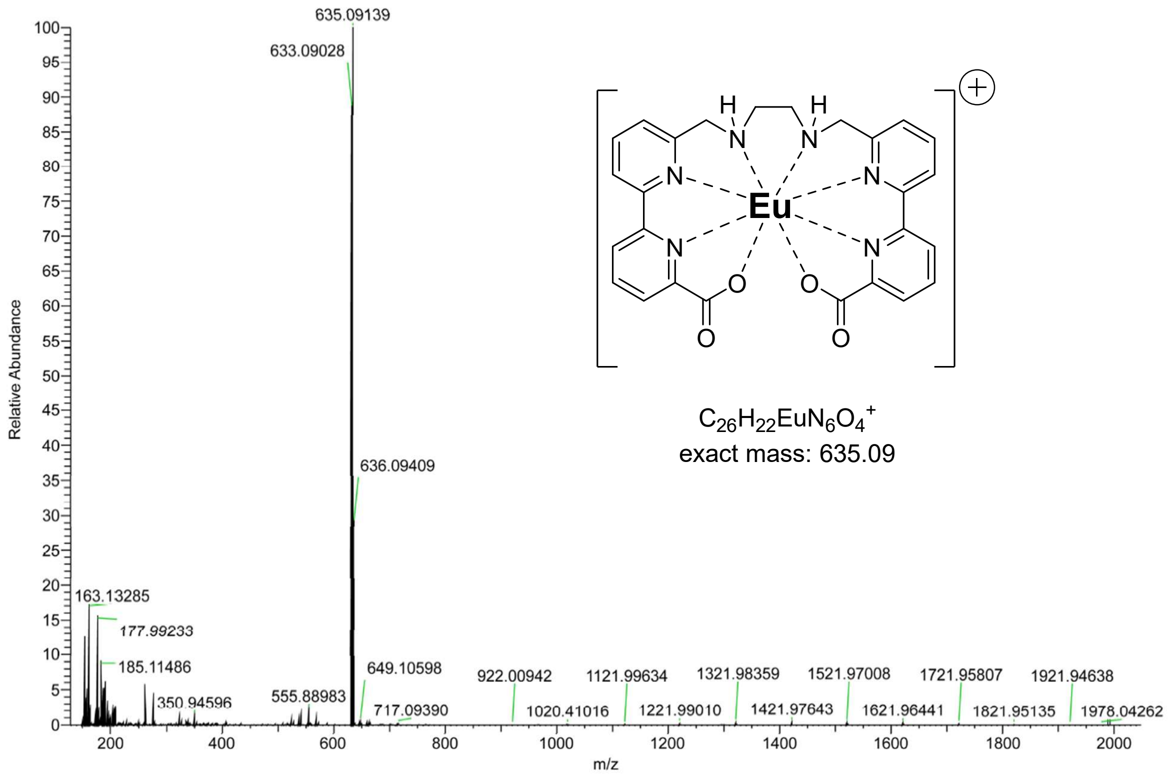

Figure S15. High resolution mass spectrum (ESI, pos. mode) of **5-Eu**.

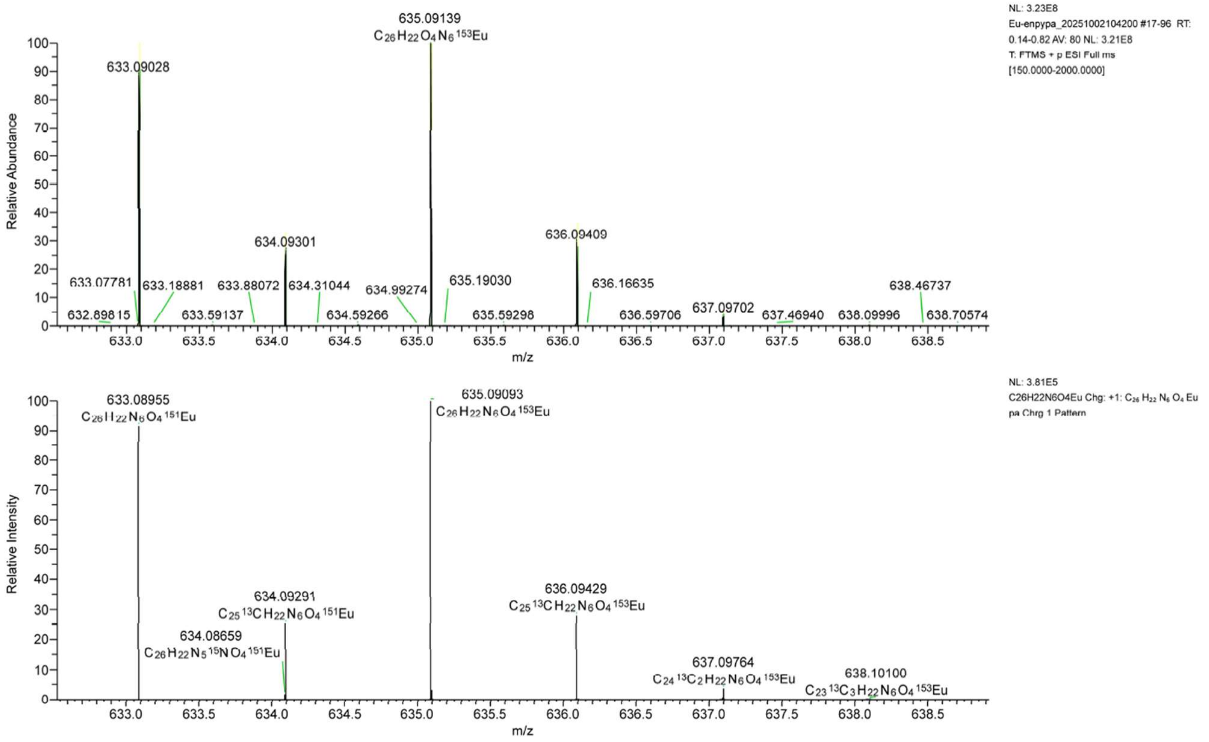

Peak Mass	Display Formula	S Fit	RDB	Delta [ppm]	Theo. mass	Rank	Combined Score	MS Cov. [%]	Pattern Cov. [%]	MSMS Matched...
635.09139	$C_{26}H_{22}O_4N_6{}^{153}Eu$	46.6859231691784	19.00	0.73	635.09093	1	96.77	99.55	99.82	(Collection)

Figure S16. Measured (top) vs. simulated (bottom) molecular ion peak of **5-Eu**.

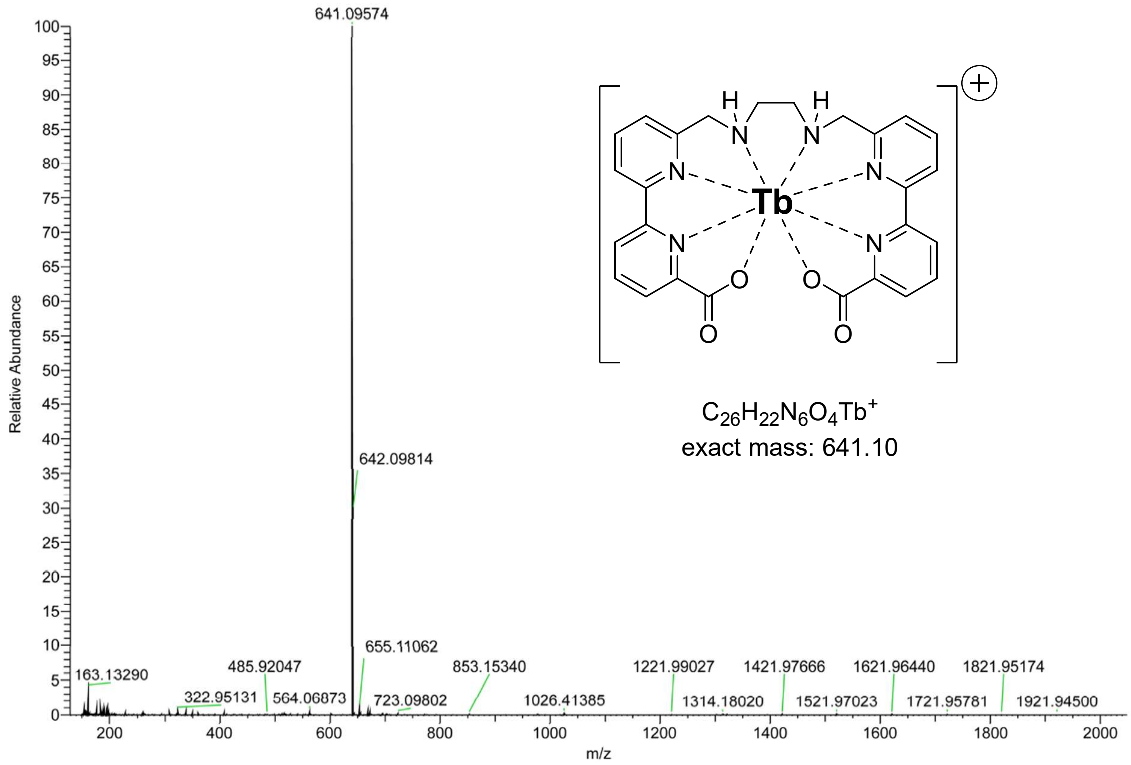

Figure S17. High resolution mass spectrum (ESI, pos. mode) of **5-Tb**.

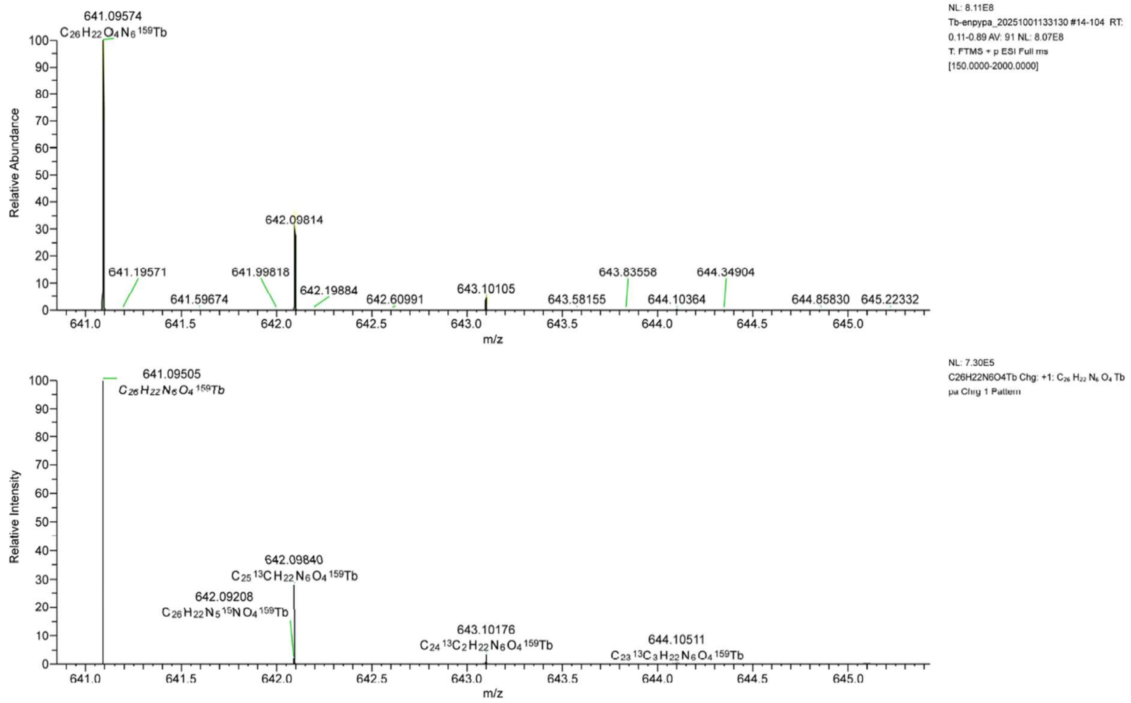

Peak Mass	Display Formula	S Fit	RDB	Delta [ppm]	Theo. mass	Rank	Combined Score	MS Cov. [%]	Pattern Cov. [%]	MSMS Matched...
641.09574	$C_{26}H_{22}O_4N_6{}^{159}Tb$	43.7325345114888	19.00	1.08	641.09505	1	96.67	99.61	99.66	(Collection)

Figure S18. Measured (top) vs. simulated (bottom) molecular ion peak of **5-Tb**.

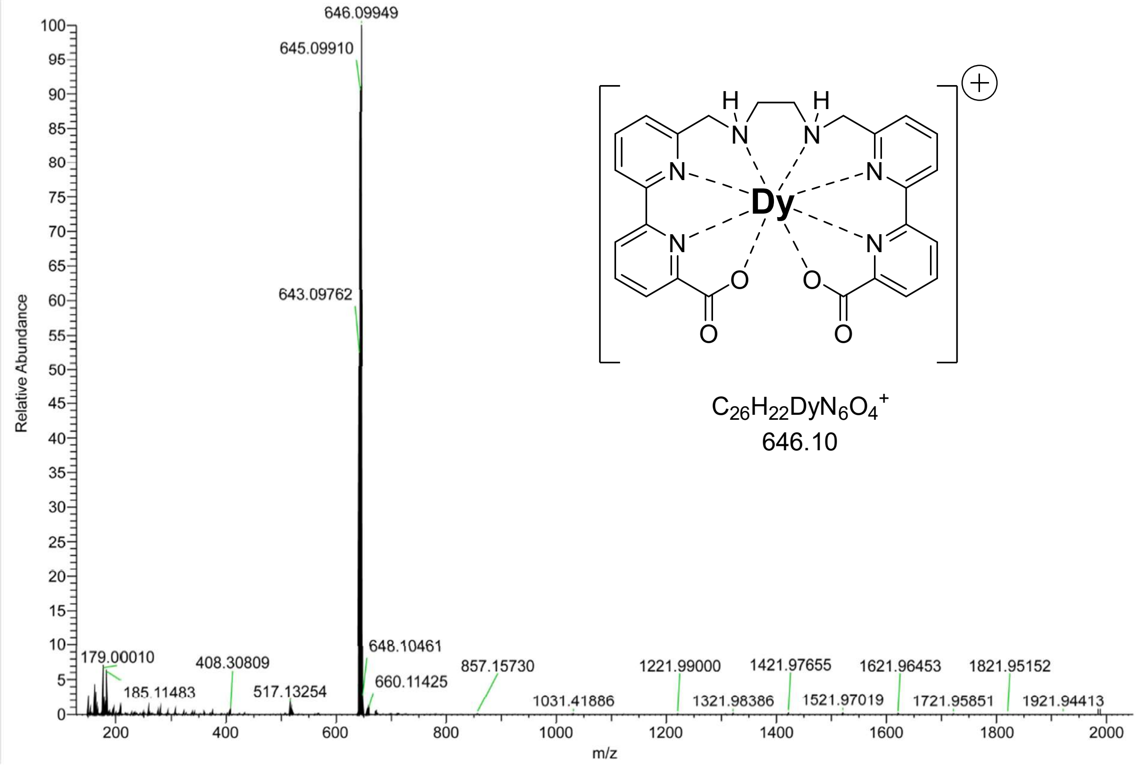

Figure S19. High resolution mass spectrum (ESI, pos. mode) of **5-Dy**.

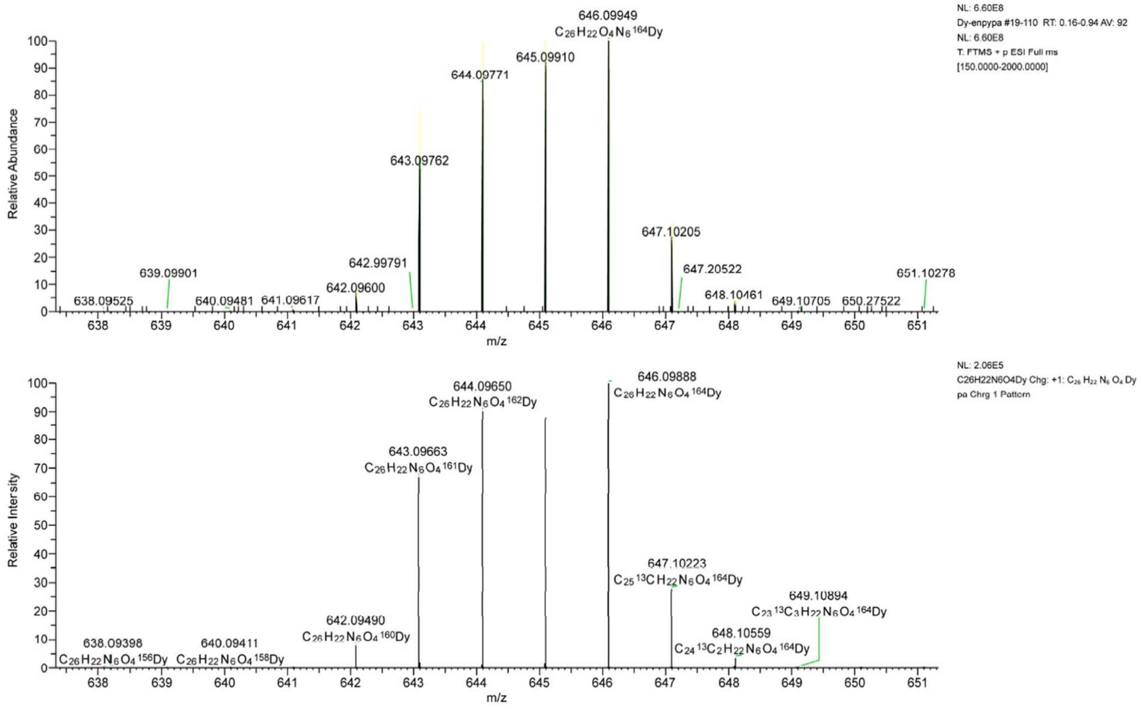

Peak Mass	Display Formula	S Fit	RDB	Delta [ppm]	Theo. mass	Rank	Combined Score	MS Cov. [%]	Pattern Cov. [%]	MSMS Matched..
646.09949	$C_{26}H_{22}O_4N_6{}^{164}Dy$	22.47524582441 75	19.00	0.95	646.09888	1	93.4	97.34	96.74	(Collection)

Figure S20. Measured (top) vs. simulated (bottom) molecular ion peak of **5-Dy**.

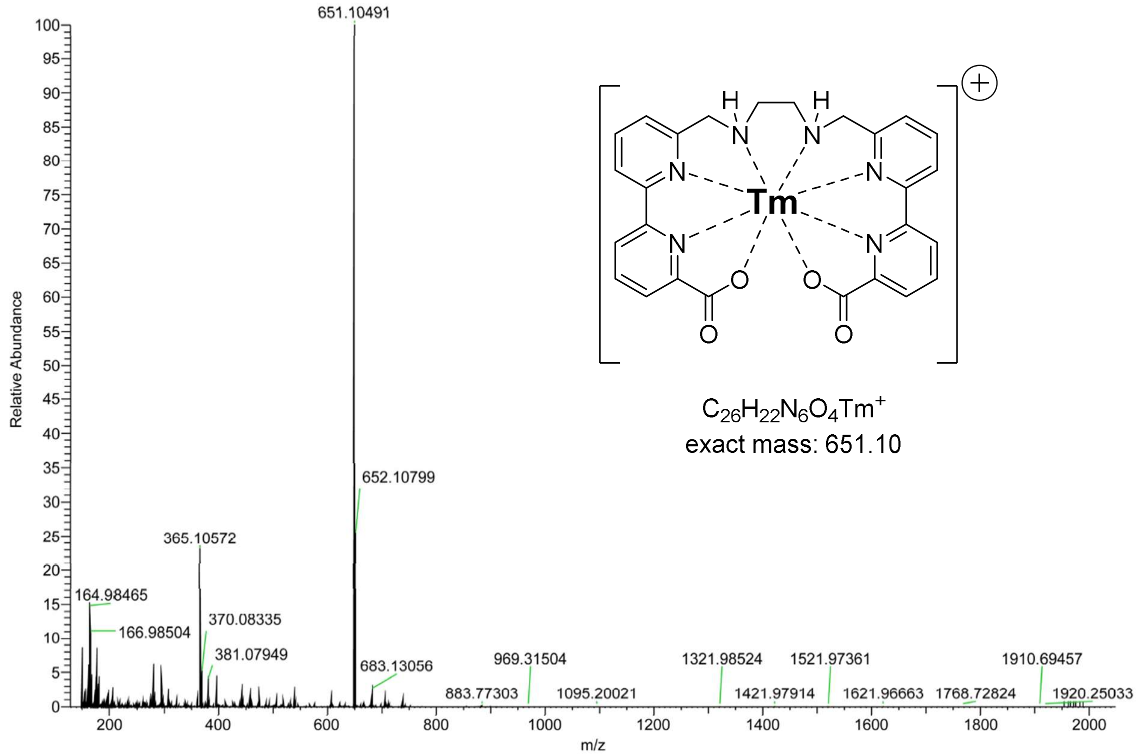

Figure S21. High resolution mass spectrum (ESI, pos. mode) of **5-Tm**.

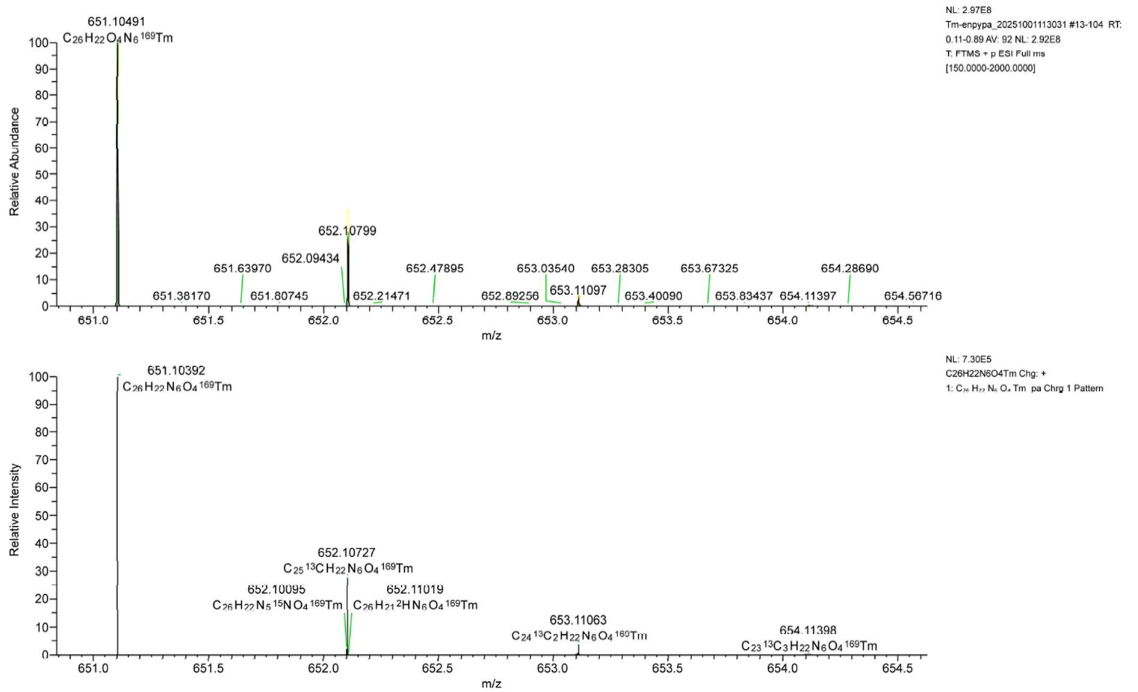

Peak Mass	Display Formula	S Fit	RDB	Delta [ppm]	Theo. mass	Rank	Combined Score	MS Cov. [%]	Pattern Cov. [%]	MSMS Matched...
651.10491	$C_{26}H_{22}O_4N_6{}^{169}Tm$	20.6740964280859	19.00	1.53	651.10392	1	93.61	97.67	96.47	(Collection)

Figure S22. Measured (top) vs. simulated (bottom) molecular ion peak of **5-Tm**.

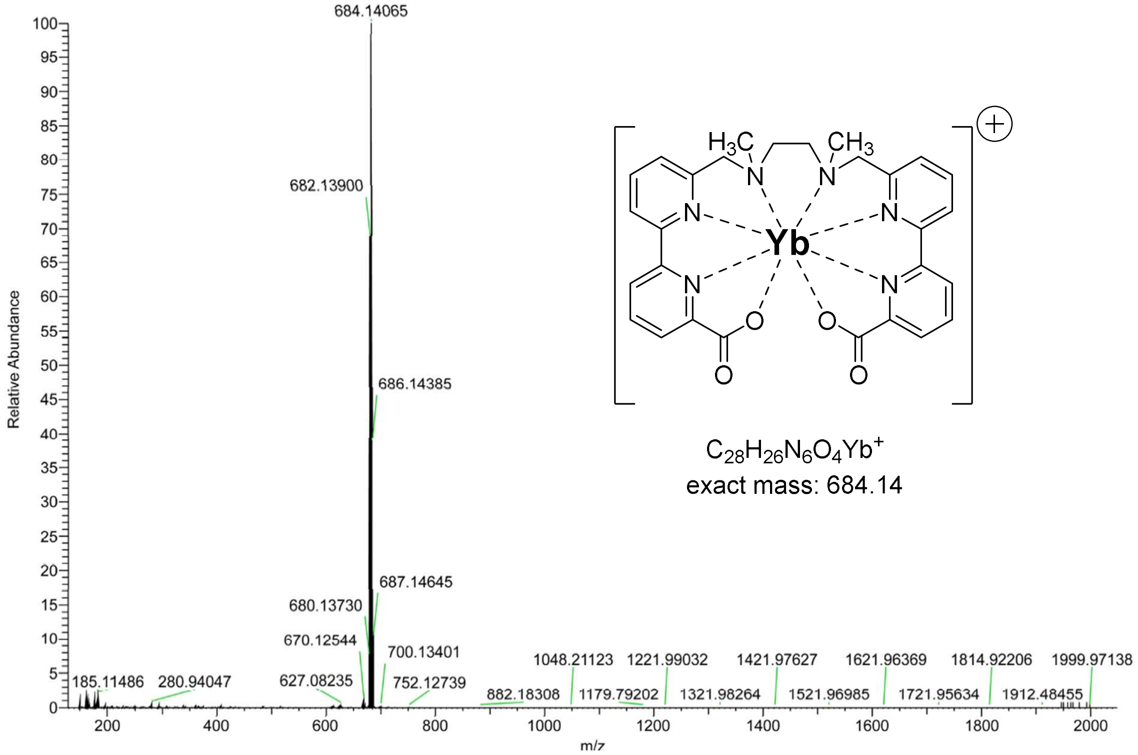

Figure S23. High resolution mass spectrum (ESI, pos. mode) of **10-Yb**.

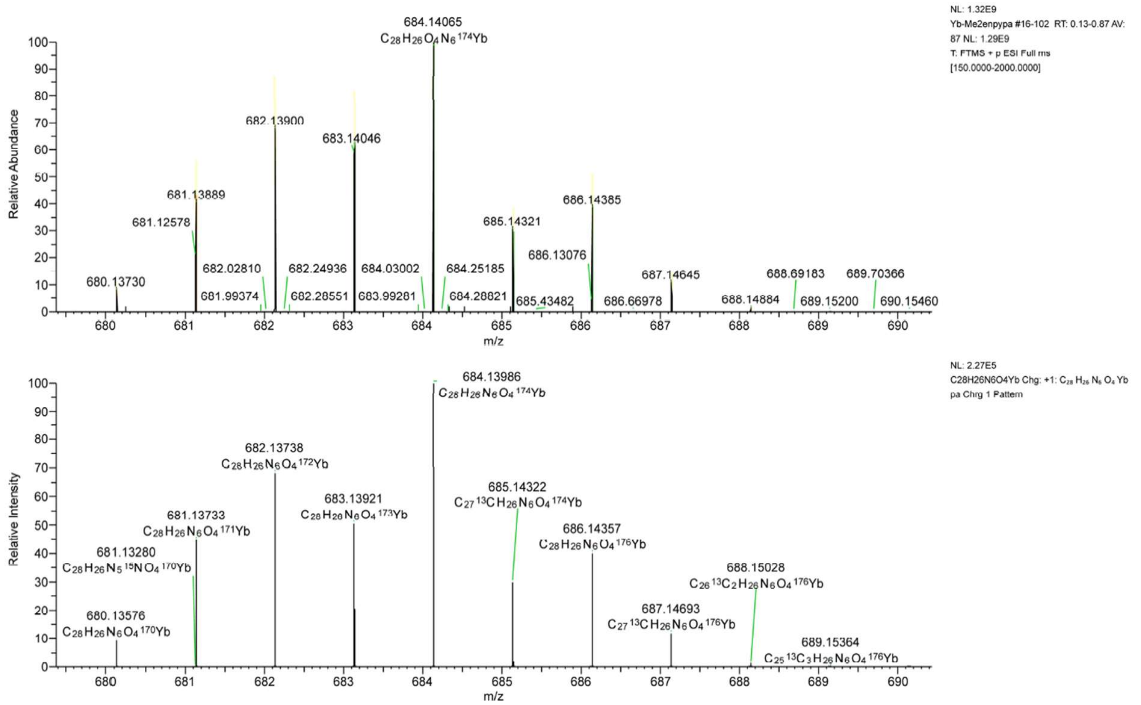

Peak Mass	Display Formula	S Fit	RDB	Delta [ppm]	Theo. mass	Rank	Combined Score	MS Cov. [%]	Pattern Cov. [%]	MSMS Matched...
684.14065	$C_{28}H_{26}O_4N_6{}^{174}Yb$	21.20154811259 82	19.00	1.15	684.13986	1	64.66	67.07	66.65	(Collection)

Figure S24. Measured (top) vs. simulated (bottom) molecular ion peak of **10-Yb**.

3 Lanthanoid-Induced NMR Shift Analysis

The paramagnetic nature of the Yb(III) ion leads to significant paramagnetic shifts in the ^{1}H NMR spectra of its complexes, as evident from comparison of the spectra of **5-Yb** and its diamagnetic analogue **5-Lu.** As the extent of the paramagnetic shifts δ_i^{para} depends on the relative spatial arrangement of the proton *i* and the paramagnetic ion, they contain structural information and can, for example, be used to experimentally validate theory-derived structural models. For any nucleus, δ_i^{para} can be extracted from the experimentally observable shifts in the ^{1}H NMR spectra of the Yb(III) ion (δ_i^{dia}) and the diamagnetic reference (δ_i^{dia}).

$$\delta_i^{para} = \delta_i^{exp} - \delta_i^{dia} = \delta_i^{pc} + \delta_i^{c}$$

Generally they consist of a contact contribution δ_i^{c} and a pseudo-contact contribution δ_i^{pc}, however, in the case of Yb(III) it has been shown that the overall shift is dominated by δ_i^{pc}, so that typically it can be assumed that $\delta_i^{para} \approx \delta_i^{pc}$.

$$\delta_i^{pc} = \left(\chi_{zz} - \frac{1}{3}Tr\chi\right)\left(\frac{3z^2 - r^2}{r^5}\right) + \left(\chi_{xx} - \chi_{yy}\right)\left(\frac{x^2 - y^2}{r^5}\right) + \chi_{xy}\left(\frac{4xy}{r^5}\right) + \chi_{xz}\left(\frac{4xz}{r^5}\right) + \chi_{yz}\left(\frac{4yz}{r^5}\right)$$

With $r = \sqrt{x^2 + y^2 + z^2}$

For any nucleus *i*, δ_i^{pc} depends on the Cartesian coordinates x, y, z of *i* relatively to the paramagnetic ion and the ion's susceptibility tensor χ in the specific coordination environment. In the principal magnetic axis system, three of the five components of the tensor χ (χ_{xy}, χ_{xz}, χ_{yz}) are zero, so that only the axial and rhombic anisotropies have to be considered.

Table S1. Experimental and calculated ^{1}H NMR shifts for **5-Yb** and **5-Lu** in D_2O at 298 K, agreement factor AF and calculated tensor components from the analysis of the lanthanoid induced shifts

Protons[a]	δ(Yb) = δ^{exp}	δ(Lu) = δ^{dia}	Experimental paramagnetic shifts ($\delta^{para} = \delta^{exp} - \delta^{dia}$)	Average calculated paramagnetic shift[b]	Calculated δ(Yb) = δ^{calc}
H1	11.21	7.74	3.47	4.98	8.45
H2	10.22	8.48	1.74	0.01	8.48
H3	5.08	8.50	-3.42	-4.59	3.91
H4	-0.32	8.24	-8.56	-6.85	1.39
H5	3.54	8.31	-4.77	-3.77	4.54
H6	8.57	8.69	-0.12	-1.64	7.05
H7	30.5	4.64	25.86	25.82	30.46
H8	8.21	4.15	4.06	4.34	8.49
AF[c]	0.13				
$\chi_{zz} - \frac{1}{3}Tr\chi$	783.56				
$\chi_{xx} - \chi_{yy}$	-411.92				

[a] According to the numbering scheme in Figure S25. Protons H9 and H10 were omitted from the analysis. [b] Average of both values calculated for the symmetry related protons. [c] The agreement factor was calculated as $AF = \sqrt{\sum_i(\delta_i^{exp} - \delta_i^{calc})^2 / \sum_i(\delta_i^{exp})^2}$.

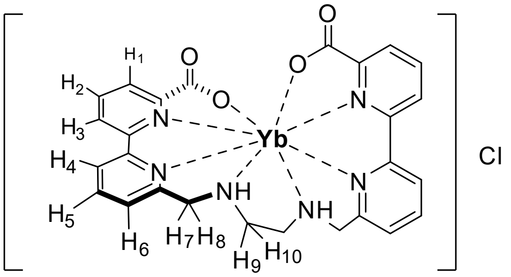

Figure S25. Numbering scheme for the protons in **5-Yb**.

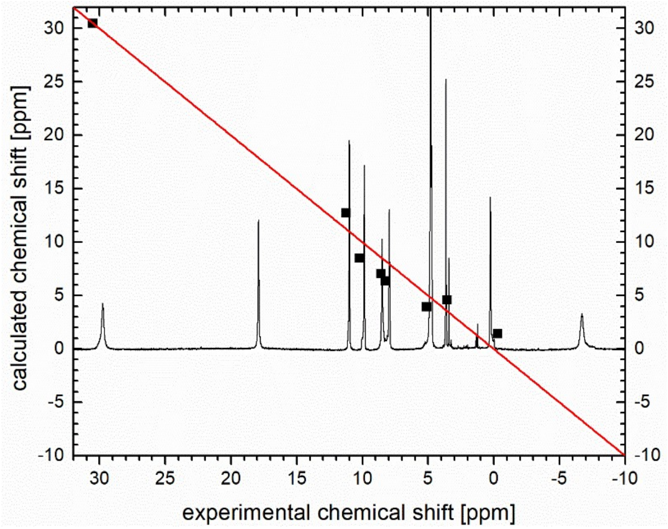

Figure S26. ^{1}H NMR (400 MHz, D_2O) spectrum of **5-Yb** and plot of experimental shifts versus those calculated by LIS analysis (see text and Table S1). The solid, diagonal line indicates where a perfectly fitting, calculated chemical shift would be situated.

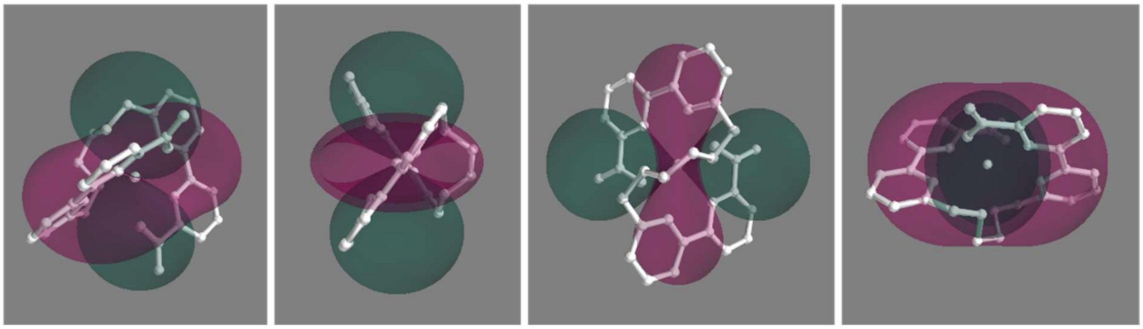

Figure S27. Illustration of the tensor of the susceptibility of the magnetic anisotropy of **5-Yb**, represented by isoshift surfaces of ±8 ppm in four different orientations.

4 Details for Quantum Chemical Calculations

CHYF calculations[S1] for **5-Yb** have been performed using the def2-TZVP basis set for H, C, N, and O,[S2] and the large-core ECP basis set for Yb with 59 electrons in the effective potential.[S3] All energies have been converged to changes of 10^{-7} Hartree in energy and 10^{-6} in the density. A grid of size 3 was used in the numerical DFT integration.[S4] Geometries were optimized to changes of 10^{-5} Hartree in energy and 10^{-4} in the gradient norm. A numerical frequency analysis was performed to confirm the obtained geometry to be stationary. All calculations have been performed using Turbomole V7.9.[S5]

Table S2. Coordinates (xyz format) for the optimized structure of **5-Yb**

N	-0.0091085	-2.5535920	-0.3167447
H	0.0633994	-2.7511895	-1.3116351
O	-0.9292642	1.9927936	-0.0795786
O	-1.9040502	3.7818690	0.8727262
C	0.3261048	0.5267518	3.3540057
C	-0.7297933	3.0620567	3.3794497
H	-1.1695799	4.0505693	3.3282010
O	4.2371377	-0.1941088	-0.8512761
C	1.1723797	-3.0998688	0.3564449
H	2.0653371	-2.7484322	-0.1686957
H	1.1801445	-4.1963192	0.3427583
C	1.2463518	-2.6023533	1.7727048
N	0.7228129	-1.4011040	2.0084814
C	0.8633989	-0.8433753	3.2206418
N	-0.1762222	1.0532428	2.2290341
C	0.3457168	1.2598797	4.5320469
H	0.7725763	0.8545324	5.4395436
C	1.5015823	-1.5170087	4.2523709
H	1.6184495	-1.0645448	5.2271701
C	2.0025938	-2.7845196	4.0178702
H	2.4965532	-3.3317868	4.8138985
C	1.8794897	-3.3402186	2.7588857
H	2.2728417	-4.3268824	2.5401345
C	-1.2396786	-3.1643712	0.2039808
H	-1.1654888	-3.1731772	1.2944040
C	-2.4428359	-2.3591731	-0.2156831
H	-2.4706993	-2.2815095	-1.3055332
N	-2.3546903	-0.9946705	0.3210758
H	-2.5681466	-1.0147440	1.3150923
C	-3.3099998	-0.1058933	-0.3457510
H	-3.3203941	0.8525691	0.1812842
H	-4.3281091	-0.5130672	-0.3306218
C	-2.8802312	0.1512982	-1.7625763
C	-3.8049019	0.4564439	-2.7470726
H	-4.8659607	0.4573210	-2.5235111
C	-3.3413685	0.7663922	-4.0111481
H	-4.0369424	1.0124598	-4.8067730
C	-1.9795732	0.7745596	-4.2510545
H	-1.6090454	1.0418747	-5.2305665
C	-1.1108613	0.4509254	-3.2183287
N	-1.5705383	0.1186372	-2.0024404
C	0.3607511	0.4867122	-3.3535786
N	1.0425152	0.2450904	-2.2255667
C	2.3759351	0.2579234	-2.2304941
C	3.0381003	-0.0190525	-0.8950390
O	2.1974072	-0.0579050	0.0877470
C	3.1076184	0.5204329	-3.3763282
H	4.1895124	0.5101757	-3.3236047
C	2.4137320	0.8035455	-4.5381967
H	2.9449664	1.0463500	-5.4528507
C	1.0274841	0.7828604	-4.5340840
H	0.4871353	1.0057233	-5.4443165
C	-0.6949740	2.2815389	2.2365609
C	-1.2373327	2.7695050	0.9081604
C	-0.1828916	2.5414449	4.5373580
H	-0.1620845	3.1283179	5.4499073
H	-1.3444169	-4.2043541	-0.1280483
H	-3.3648524	-2.8604465	0.1031837
Yb	0.0000000	0.0000000	0.0000000

5 References

[S1] Holzer, C.; Franzke, Y. J.; A General and Transferable Local Hybrid Functional for Electronic Structure Theory and Many-Fermion Approaches. *J. Chem. Theory Comput.* **2025**, *21*, 202.

[S2] Weigend, F.; Ahlrichs, R.; Balanced basis sets of split valence, triple zeta valence and quadruple zeta valence quality for H to Rn: Design and assessment of accuracy. *Phys. Chem. Chem. Phys.* **2005**, *7*, 3297.

[S3] Dolg, M.; Stoll, H.; Savin, A.; Preuss, H.; Energy-adjusted pseudopotentials for the rare earth elements. *Theoret. Chim. Acta* **1989**, *75*, 173.

[S4] Treutler, O.; Ahlrichs, R.; Efficient molecular numerical integration schemes. *J. Chem. Phys.* **1995**, *102*, 346.

[S5] Franzke, Y. J.; Holzer, C.; Andersen, J. H.; Begušić, T.; Bruder, F.; Coriani, S.; Della Sala, F.; Fabiano, E.; Fedotov, D. A.; Fürst, S.; Gillhuber, S.; Grotjahn, R.; Kaupp, M.; Kehry, M.; Krstić, M.; Mack, F.; Majumdar, S.; Nguyen, B. D.; Parker, S. M.; Pauly, F.; Pausch, A.; Perlt, E.; Phun, G. S.; Rajabi, A.; Rappoport, D.; Samal, B.; Schrader, T.; Sharma, M.; Tapavicza, E.; Treß, R. S.; Voora, V.; Wodyński, A.; Yu, J. M.; Zerulla, B.; Furche, F.; Hättig, C.; Sierka, M.; Tew, D. P.; Weigend, F.; TURBOMOLE: Today and Tomorrow. *J. Chem. Theory Comput.* **2023**, *19*, 6859.
